# Intestinal microbiota shapes gut physiology and regulates enteric neurons and glia

**DOI:** 10.1186/s40168-021-01165-z

**Published:** 2021-10-26

**Authors:** Fernando A. Vicentini, Catherine M. Keenan, Laurie E. Wallace, Crystal Woods, Jean-Baptiste Cavin, Amanda R. Flockton, Wendy B. Macklin, Jaime Belkind-Gerson, Simon A. Hirota, Keith A. Sharkey

**Affiliations:** 1 Hotchkiss Brain Institute, University of Calgary, Calgary, AB T2N 4N1 Canada; 2 Snyder Institute for Chronic Diseases, University of Calgary, Calgary, AB T2N 4N1 Canada; 3 Inflammation Research Network, University of Calgary, Calgary, AB T2N 4N1 Canada; 4 Department of Physiology and Pharmacology, Cumming School of Medicine, University of Calgary, 3330 Hospital Drive NW, Calgary, AB T2N 4N1 Canada; 5 Department of Pediatrics, Section of Gastroenterology, Hepatology and Nutrition, University of Colorado, Aurora, CO 80045 USA; 6 Department of Cell and Developmental Biology, University of Colorado School of Medicine, Aurora, CO 80045 USA; 7 Neurogastroenterology and Motility Program, Digestive Health Institute, Children’s Hospital Colorado, Aurora, CO 80045 USA; 8 Alberta Children’s Hospital Research Institute, University of Calgary, Calgary, AB T2N 4N1 Canada

**Keywords:** Enteric nervous system, Gastrointestinal motility, Short-chain fatty acids, LPS, Enteric glia, Myenteric plexus, Submucosal plexus

## Abstract

**Background:**

The intestinal microbiota plays an important role in regulating gastrointestinal (GI) physiology in part through interactions with the enteric nervous system (ENS). Alterations in the gut microbiome frequently occur together with disturbances in enteric neural control in pathophysiological conditions. However, the mechanisms by which the microbiota regulates GI function and the structure of the ENS are incompletely understood. Using a mouse model of antibiotic (Abx)-induced bacterial depletion, we sought to determine the molecular mechanisms of microbial regulation of intestinal function and the integrity of the ENS. Spontaneous reconstitution of the Abx-depleted microbiota was used to assess the plasticity of structure and function of the GI tract and ENS. Microbiota-dependent molecular mechanisms of ENS neuronal survival and neurogenesis were also assessed.

**Results:**

Adult male and female Abx-treated mice exhibited alterations in GI structure and function, including a longer small intestine, slower transit time, increased carbachol-stimulated ion secretion, and increased intestinal permeability. These alterations were accompanied by the loss of enteric neurons in the ileum and proximal colon in both submucosal and myenteric plexuses. A reduction in the number of enteric glia was only observed in the ileal myenteric plexus. Recovery of the microbiota restored intestinal function and stimulated enteric neurogenesis leading to increases in the number of enteric glia and neurons. Lipopolysaccharide (LPS) supplementation enhanced neuronal survival alongside bacterial depletion, but had no effect on neuronal recovery once the Abx-induced neuronal loss was established. In contrast, short-chain fatty acids (SCFA) were able to restore neuronal numbers after Abx-induced neuronal loss, demonstrating that SCFA stimulate enteric neurogenesis in vivo.

**Conclusions:**

Our results demonstrate a role for the gut microbiota in regulating the structure and function of the GI tract in a sex-independent manner. Moreover, the microbiota is essential for the maintenance of ENS integrity, by regulating enteric neuronal survival and promoting neurogenesis. Molecular determinants of the microbiota, LPS and SCFA, regulate enteric neuronal survival, while SCFA also stimulates neurogenesis. Our data reveal new insights into the role of the gut microbiota that could lead to therapeutic developments for the treatment of enteric neuropathies.

Video abstract

**Supplementary Information:**

The online version contains supplementary material available at 10.1186/s40168-021-01165-z.

## Background

The gastrointestinal (GI) tract is regulated by finely tuned interactions of its cellular components, controlled by an intrinsic nervous system, the enteric nervous system (ENS) [1, 2]. The ENS is composed of two ganglionated plexuses, the myenteric and submucosal plexuses, containing neurons and enteric glial cells (EGC), connected by a dense network of nerve fibers and glial processes within the gut wall [1]. The ENS regulates GI motility, fluid secretion/absorption, immune function, mucosal growth and integrity, and intestinal permeability [1–5]. Structural and functional alterations in the ENS are linked to GI diseases, including irritable bowel syndrome and inflammatory bowel disease [6, 7]. These debilitating conditions are increasing in incidence globally and have an enormous socioeconomic impact [7, 8].

The intestinal microbiota plays a fundamental role as a determinant of human health and is a critical factor that regulates GI physiology and pathophysiology [9–11]. Early-life alterations in the intestinal microbiota cause changes in gut physiology, which can lead to long-lasting effects on overall health [12–15]. More recently, there has been a focus on understanding the role of the microbiota on GI function in the mature, adult animal, including the microbiota’s influence on the structure and function of the ENS [16–27]. With the widespread use of probiotics [28], and the increasing application of fecal microbiota transplant therapy [29], it is evident that a better understanding of the impact of host-microbe interactions on GI physiology is required. The molecular mechanisms responsible for the interactions between the intestinal microbiota and the ENS are an area of active investigation. Two classes of molecules have emerged as playing important roles: ligands for toll-like receptors (TLR) and short-chain fatty acids (SCFA).

TLRs recognize microbial-associated molecular patterns (MAMPs) and are involved in host defense mechanisms of the innate immune system [30]. Recently, the role of TLRs has expanded beyond classical immune responses [31], with receptor expression being identified in other cell types, including in neurons of the ENS [18, 25, 32, 33]. Recent reports suggest that TLRs regulate neuronal survival and neurogenesis [18, 25, 32]. MAMPs, such as lipopolysaccharide (LPS), are expressed by many classes of commensal enteric bacteria and are likely involved in the functional interactions of the microbiota and the ENS. In addition to MAMPs, bacterial products have also been shown to influence GI function and the structure of the ENS [34, 35]. SCFA are well-characterized bacterial products that have been demonstrated to affect GI physiology, through modulation of 5-hydroxytryptamine (5-HT; serotonin) [24, 36] and other mechanisms [37].

The role of these microbial determinants in regulating GI function and the structural integrity of the ENS in adult animals remains to be fully elucidated. Our goals were to determine the role of the microbiota and their molecular mediators (i.e., LPS and SCFA) on GI motility, intestinal permeability, fecal output, and the structural organization of the ENS, using a mouse model of antibiotic (Abx)-induced bacterial depletion. We sought to determine whether alterations in GI function and the ENS could be reversed upon reconstitution of the intestinal microbiota over the course of a 3-week Abx washout period. To determine the role of LPS and SCFA in the regulation of gut function and ENS structure, we supplemented Abx-treated mice with either LPS or SCFA at different timepoints to investigate the impact of these key host-microbe interactions.

## Results

### Abx-induced depletion of the intestinal microbiota alters the structure and function of the gut in a sex-independent manner

**Fig. 1:**
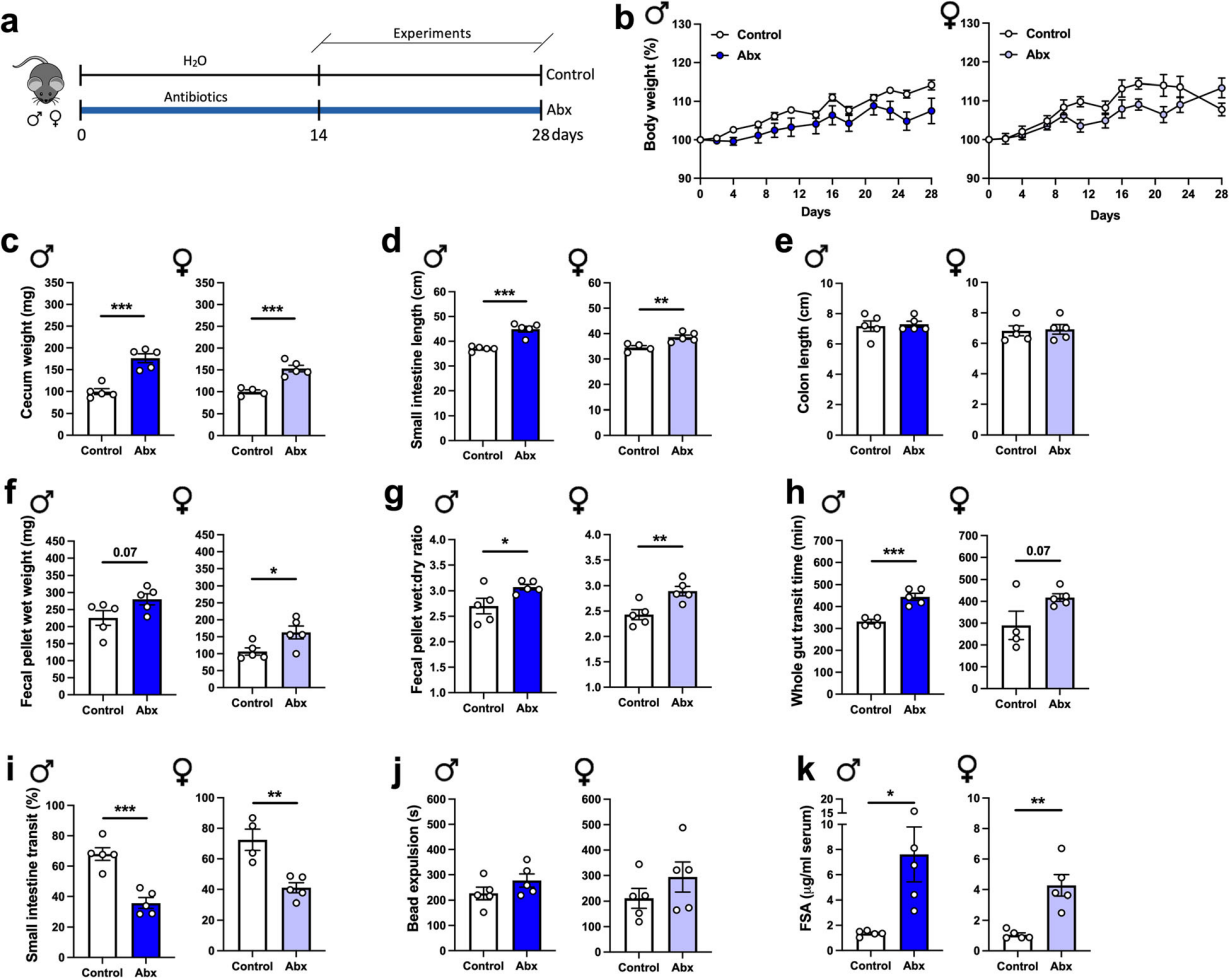
Antibiotic (Abx) treatment alters GI structure, delays transit, and increases intestinal permeability in adult mice in a sex-independent manner. **a** Adult mice of both sexes were treated with a combination of antibiotics for at least 14 days in the drinking water prior to experiments (Abx group). The antibiotic regimen consisted of ampicillin (1 g/L), neomycin (1 g/L), vancomycin (0.5 g/L), and metronidazole (1 g/L), administered as described in “Methods.” The control group received regular water. Experiments were performed between 14 and 28 days. **b** Body weight variation over the course of the experiment (two-way ANOVA). **c–e** Intestinal anatomical alterations observed in Abx mice; **c** cecal wet weight, **d** small intestinal length, and **e** colon length. **f** Fecal pellet wet weight and **g** wet:dry ratio of feces measured after a 1-h novel environment stress. **h** Whole gut transit time. **i** Small intestinal transit distance (as a % of total intestinal length) measured 15 min after gavage with dye. **j** Distal colonic motility measured by bead expulsion time. **k** Intestinal permeability assessed by fluorescein-5-6-sulfonic acid (FSA) concentration in the serum 4 h after gavage with FSA. Data are expressed as mean ± SEM. *n* = 4–5. *p < 0.05, **p < 0.01, ***p < 0.001; Student’s *t* test

We first examined the effects of depleting the intestinal microbiota on GI morphology and physiology. We treated mice with an Abx regimen (Fig. 1a) that effectively reduces the enteric microbiota by over 99%, as we have shown previously [38]. No alterations in body weight were observed over the course of Abx treatment (Fig. 1b). Interestingly, anatomical alterations were observed in both sexes in the cecum (Fig. 1c) and small intestine (Fig. 1d), but not in the colon (Fig. 1e), where the absolute number of bacteria are most abundant [39]. Abx-treated mice exhibited larger ceca (Fig. 1c) and an increase in small intestinal length (Fig. 1d) compared to non-Abx control mice. To determine whether the increased small intestinal length was due to tissue elongation, and not an effect of reduced contractile tone, tissues from both groups were treated with nifedipine, an L-type voltage-gated calcium channel blocker, to induce maximal muscle relaxation. Despite inducing relaxation with nifedipine, small intestinal tissues from Abx-treated mice were still significantly longer than control tissues (Figure S1), suggesting that the intestine lengthens to compensate for the loss of the microbiota.

To evaluate the effects of Abx treatment on intestinal function, we first assessed the fecal pellet output during a novel environment stress test. We also measured the water content in the expelled feces to detect any net alterations in secretion and/or absorption. In both male and female mice, Abx treatment induced higher fecal pellet wet weight compared to the control group (Fig. 1f), suggesting an enhanced response to stress in Abx-treated mice. Additionally, an increase in the fecal water content was observed in Abx-treated mice (Fig. 1g), suggesting changes in water absorption and/or secretion. Next, we evaluated GI motility using three different approaches. We found that GI motility was slowed in Abx-treated mice, indicated by an increase in whole gut transit time (Fig. 1h). To determine the origin(s) of the altered whole gut transit, we performed tests to evaluate small intestinal transit and distal colonic propulsion. Small intestinal motility, assessed by measuring dye migration along the intestine, was significantly reduced in Abx-treated mice (Fig. 1i). However, in the distal colon, we observed no differences in bead expulsion time between Abx-treated and control groups (Fig. 1j). No sex-dependent changes in motility were observed in Abx-treated mice (Fig. 1h–j).

Next, we assessed intestinal barrier function by measuring fluorescein-5-6-sulfonic acid (FSA) leakage into serum [27]. Four hours following oral gavage, FSA was measured in the serum as an index of intestinal permeability. Abx-treated mice of both sexes exhibited increased serum FSA compared to control mice, suggesting that depletion of the intestinal microbiota increased intestinal permeability (Fig. 1k).

In summary, intestinal microbial depletion altered gut morphology, motility, and permeability in both male and female animals.

### Indiscriminate loss of enteric neurons is observed after depletion of gut bacteria, triggering enteric neurogenesis in the myenteric plexus

**Fig. 2:**
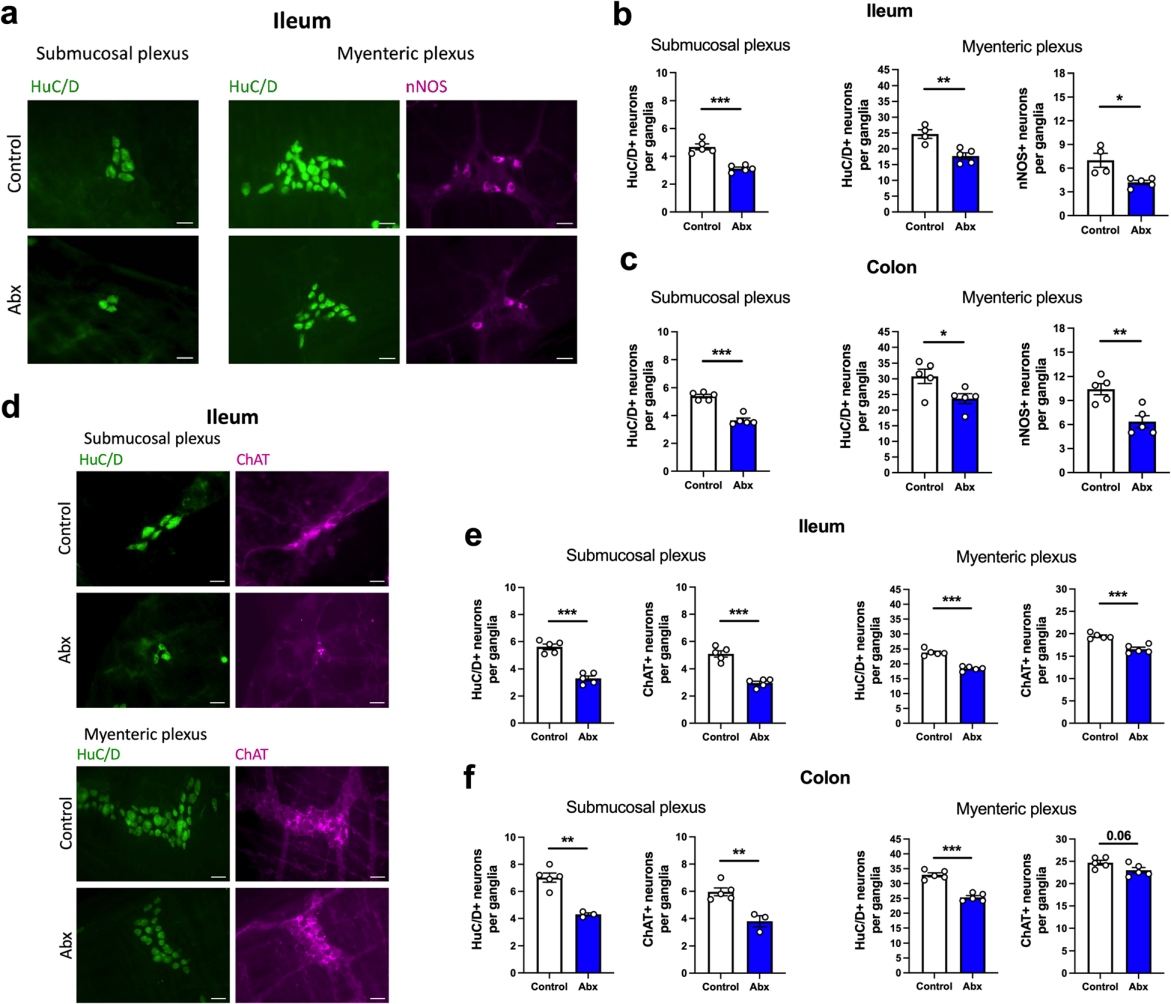
Depletion of gut bacteria is associated with an indiscriminate neuronal loss, affecting both nNOS^+^ nitrergic and ChAT^+^ cholinergic subpopulations. **a** Representative immunofluorescent images of ganglia in the submucosal and myenteric plexuses: HuC/D^+^ (green) and nNOS^+^ (magenta) neurons in the ileum of control and antibiotic (Abx)-treated mice. Scale bar: 30 μm. **b** Number of HuC/D^+^ neurons in both submucosal (left panel) and myenteric (middle panel) plexuses, and the number of nNOS^+^ neurons (right panel) in the myenteric plexus of the ileum. **c** Number of HuC/D^+^ neurons in both submucosal (left panel) and myenteric (middle panel) plexuses, and the number of nNOS^+^ neurons (right panel) in the myenteric plexus of the colon. **d** Representative immunofluorescent images of ganglia in the submucosal and myenteric plexuses: HuC/D^+^ (green) and ChAT^+^ (magenta) neurons in the ileum. Scale bar: 30 μm. **e**, **f** Number of HuC/D^+^ and ChAT^+^ neurons in both submucosal (left panels) and myenteric (right panels) in the **e** ileum and **f** colon. Data are expressed as mean ± SEM. *n* = 3–5. *p < 0.05, **p < 0.01, ***p < 0.001; Student’s *t* test

After observing alterations in GI morphology and function following Abx treatment (Fig. 1), we sought to determine if these events were associated with neuroanatomical changes in the ENS. Since we observed similar responses in male and female mice regarding Abx-induced alterations in GI function, we performed most of the remaining experiments in male mice. We first evaluated the neuroanatomical changes in two distinct intestinal regions, the distal ileum and proximal colon, analyzing both submucosal and myenteric plexuses (Fig. 2 and Figure S2). Strikingly, a loss of HuC/D^+^ neurons in Abx-treated mice was observed in both the ileum (Fig. 2b, e) and the colon (Fig. 2c, f), in the submucosal and myenteric plexuses. This loss of enteric neurons was accompanied by a reduction in the Tuj1^+^ neuronal fiber density (Figure S3a-b). By evaluating the two major subpopulations of enteric neurons, the nNOS^+^ nitrergic and the ChAT^+^ cholinergic neurons, we observed a loss of both the nNOS^+^ neurons in the myenteric plexus (Fig. 2b, c; nNOS^+^ neurons are virtually absent in the submucosal plexus) and ChAT^+^ neurons in the submucosal and myenteric plexuses (Fig. 2e, f). To further explore the effect of Abx treatment in subpopulations of enteric neurons, we evaluated the impact of bacterial depletion on calretinin (CALR^+^) neurons (Figure S3c). After Abx treatment, CALR^+^ neurons were reduced in the ileal submucosal and myenteric plexuses, and in the colonic submucosal plexus, but, interestingly, not in the colonic myenteric plexus (Figure S3d).

**Fig. 3:**
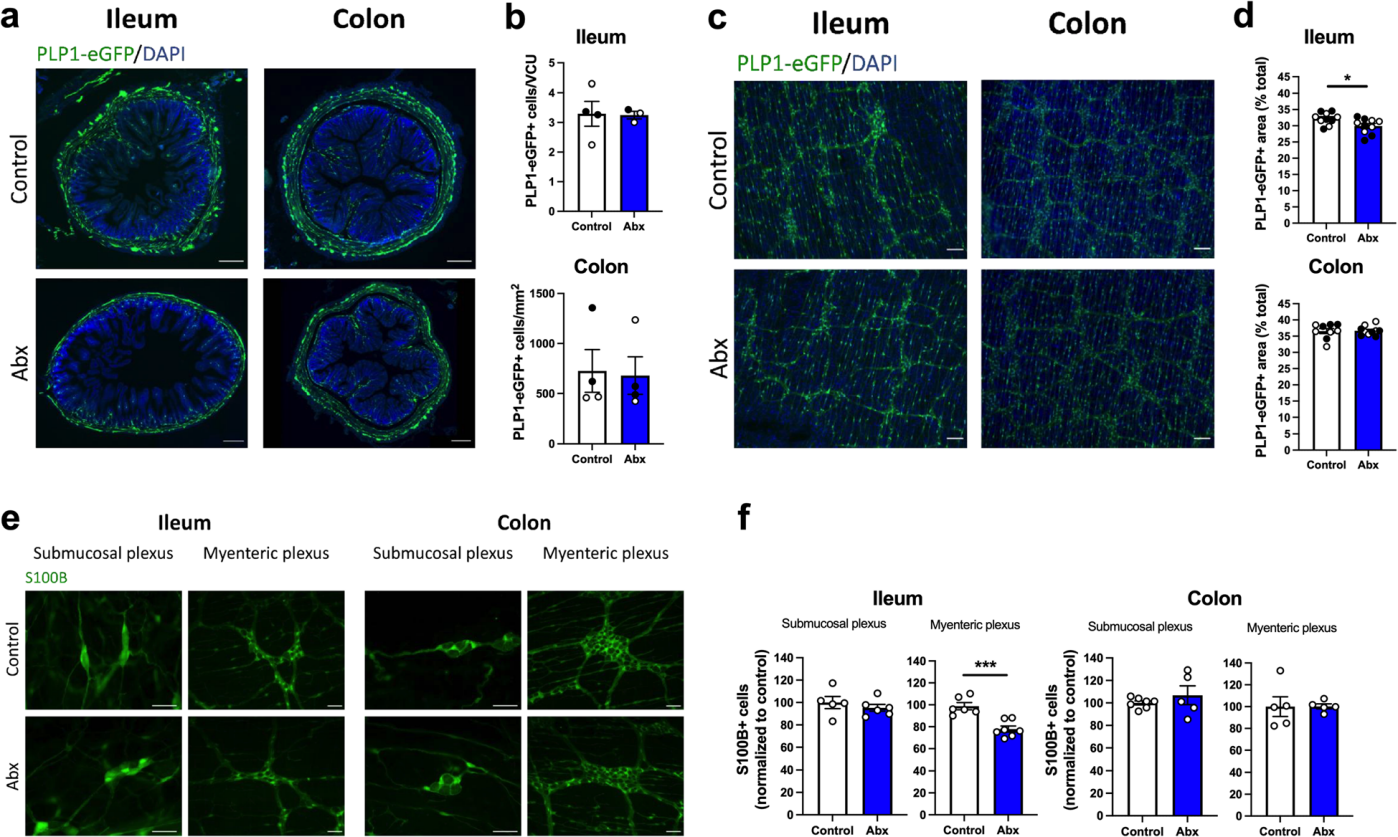
Antibiotic (Abx) treatment has regional impact on enteric glia, affecting the ileal myenteric plexus. **a** Cross sections of the intestine showing PLP1^+^ enteric glial cell (EGC) bodies and projections (green). Cross sections were counterstained with DAPI. Scale bar: 200 μm. **b** Number of PLP1-eGFP^+^ cell bodies in the mucosa in both ileum and colon. **c** Whole-mount preparations showing the myenteric plexus with PLP1-eGFP^+^ EGC (green). Preparations were counterstained with DAPI. Scale bar: 100 μm. **d** PLP1-eGFP^+^ area in whole-mount preparations of the myenteric plexus in both ileum and colon. **e** Immunofluorescent images of S100B^+^ EGC (green) in the submucosal and myenteric plexus of both ileum and colon. Scale bar: 50 μm. **f** Relative number of S100B^+^ EGC in the ileum and colon in the submucosal and the myenteric plexus. Data are expressed as mean ± SEM. **b** *n* = 3–4, mix of male and female mice. **d** *n* = 9–10, mix of male and female mice, two different cohorts were used , shown with black and white dots. **f** *n* = 5–7, male mice. Male mice are shown using white dots, female mice are shown using black dots. *p < 0.05, ***p < 0.001; Student’s *t* test. EGC, enteric glial cells; VCU, villus-crypt unit

Additionally, we analyzed the effects of Abx treatment on enteric glia. We used two approaches to identify the EGC in our experiments (Fig. 3a, e), a PLP1-eGFP-expressing mouse [40], and the immunohistochemical expression of S100B; both well-established EGC markers [41, 42]. We observed a complete overlap in the distribution of PLP1 and S100B expression (Figure S4), supporting their validity as markers of EGC. In PLP1-eGFP mice, we observed no alterations in the number of EGC cell bodies in the mucosa after the Abx treatment in either the ileum or colon (Fig. 3b). However, we detected a regional effect of intestinal microbial depletion on EGC in whole-mount preparations. A reduction in the PLP1-eGFP^+^ area (Fig. 3d), and a reduction in EGC number (Fig. 3f) were observed in the myenteric plexus in the ileum, but not the colon. The total number of EGC was not altered in the submucosal plexus of either region (Fig. 3f). Together, our data suggest a general loss of neurons in all regions analyzed after Abx-treatment, affecting neuronal subpopulations indiscriminately, and a reduction in the EGC population in the ileal myenteric plexus.

**Fig. 4:**
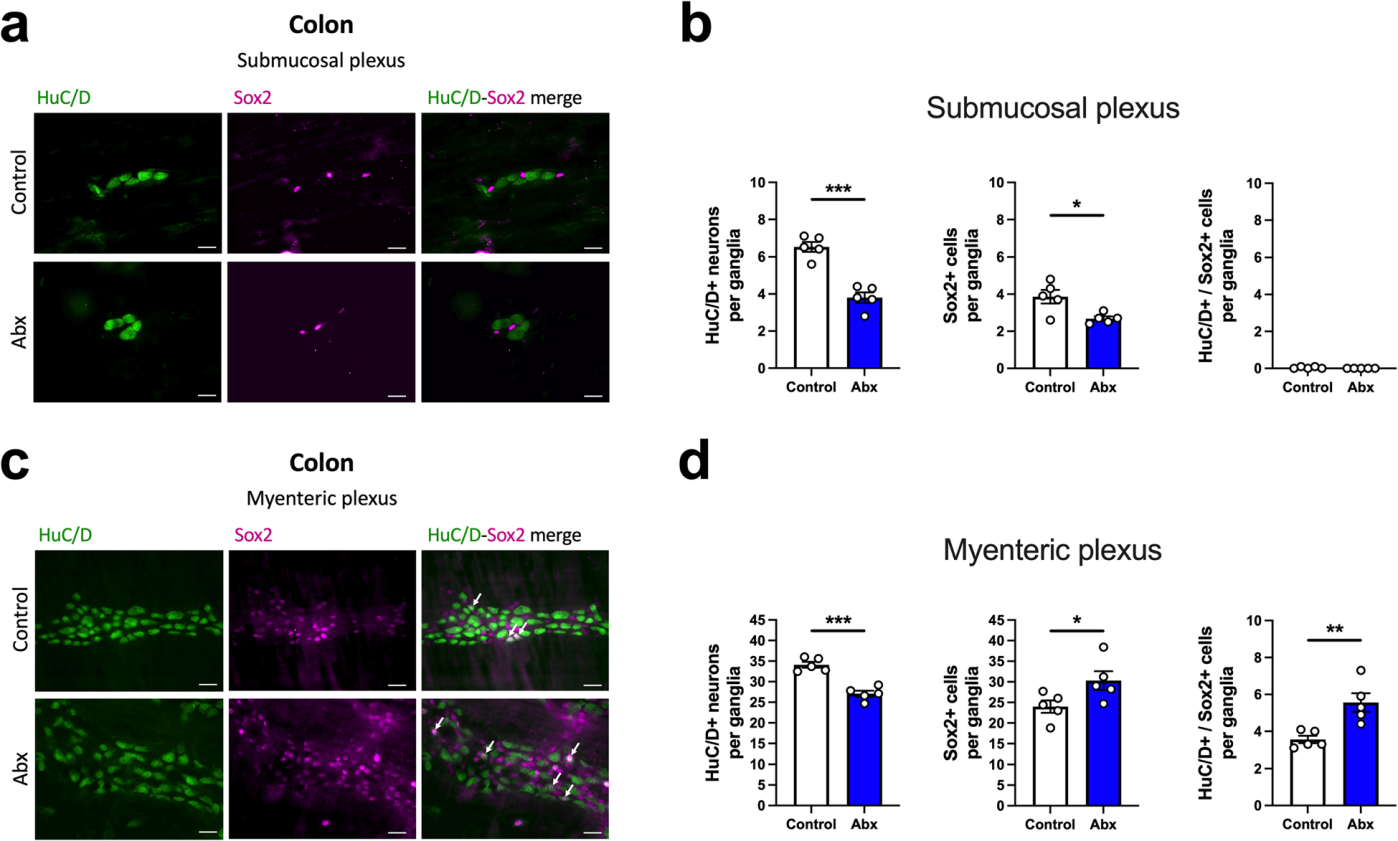
Depletion of gut microbiota stimulates neuronal Sox2 expression in the colonic myenteric plexus. **a** Representative immunofluorescent images of ganglia in the colonic submucosal plexus: HuC/D^+^ (green) and Sox2^+^ (magenta) cells. Scale bar: 30 μm. **b** Number of HuC/D^+^ neurons (left panel), Sox2^+^ cells (middle panel), and double-labelled HuC/D^+^/Sox2^+^ neurons (right panel) in the submucosal plexus of the proximal colon. **c** Representative immunofluorescent images of ganglia in the colonic myenteric plexus: HuC/D^+^ (green) and Sox2^+^ (magenta) cells. Arrows represent double-labelled HuC/D^+^/Sox2^+^ neurons. Scale bar: 30 μm. **d** Number of HuC/D^+^ neurons (left panel), Sox2^+^ cells (middle panel), and double-labelled HuC/D^+^/Sox2^+^ neurons (right panel) in the myenteric plexus of the proximal colon. Data are expressed as mean ± SEM. *n* = 5. *p < 0.05, **p < 0.01, ***p < 0.001; Student’s *t* test

We next tested the hypothesis that the broad loss of neurons in the ENS after Abx treatment would trigger a compensatory response, potentially leading to increased enteric neurogenesis [43, 44]. Sox2 is a transcription factor known as a marker of ENS progenitor cells and is largely expressed by EGC in the adult animal [45, 46]. Thus, we used HuC/D^+^/Sox2^+^ double-labeling to evaluate the presence of Sox2-expressing neurons (Fig. 4), a putative marker of enteric neurogenesis [45, 46]. We observed that the reduction of HuC/D^+^ neuronal numbers in the Abx-treated animals was accompanied by a reduction in Sox2^+^ cells in the colonic submucosal plexus (Fig. 4b). Surprisingly, we did not detect any HuC/D^+^/Sox2^+^ neurons in the submucosal plexus (Fig. 4b). In contrast, the decreased number of HuC/D^+^ neurons in the colonic myenteric plexus of Abx-treated animals was followed by an increase in Sox2^+^ cells and HuC/D^+^/Sox2^+^ neurons (Fig. 4d), indicative of increased neurogenesis in the colonic myenteric plexus of the microbiota-depleted mouse. Further, we tested whether cell proliferation was evident in the ENS by evaluating ileal and colonic sections stained for Ki67, a marker of cell proliferation. As expected, we observed Ki67^+^ cells at the base of the intestinal epithelial crypts, but we observed no positive staining in the ENS (Figure S5a). Moreover, we tested whether Abx treatment would lead to higher incorporation of 5-ethynyl-2′-deoxyuridine (EdU) into enteric cells, demonstrating de novo DNA synthesis. We did not detect positive EdU incorporation/staining in the ENS (Figure S5b). Thus, Abx treatment triggered region-specific increased expression of Sox2 in neurons, which is indicative of enteric neurogenesis; however, no markers of proliferation were detected in enteric neurons.

### Spontaneous recolonization of bacteria restores altered GI physiology and ENS neuronal loss

**Fig. 5:**
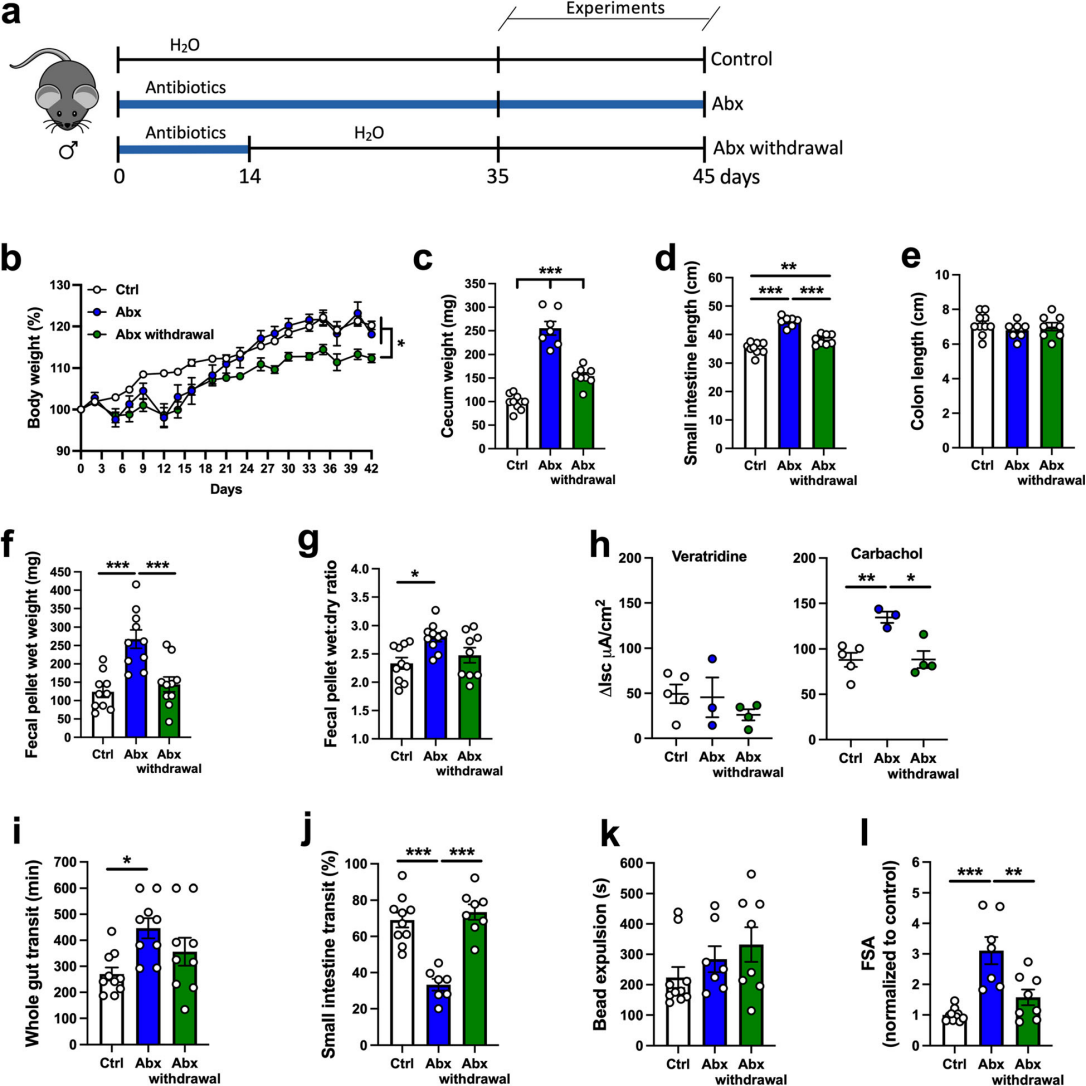
Alterations in GI function after antibiotic (Abx) treatment are restored by spontaneous microbial recolonization. **a** Male mice received Abx for 14 days and were allowed 21 days on regular drinking water for spontaneous microbiota reconstitution (Abx withdrawal). **b** Body weight change over the course of the experiment (two-way ANOVA, followed by Tukey’s multiple comparison test). **c–e** Intestinal anatomical parameters; **c** cecal weight, **d** small intestinal length, and **e** colon length. **f** Fecal pellet wet weight and **g** wet:dry ratio of the feces measured after a 1-h novel environment stress. **h** Ion transport evaluated in ileal preparations mounted in Ussing chambers after stimulation with veratridine (10 μM) or carbachol (100 μM). **i** Whole gut transit time. **j** Small intestinal transit distance (as a % of total intestinal length) measured 15 min after gavage with dye. **k** Distal colonic motility measured by bead expulsion time. **l** Intestinal permeability assessed by fluorescein-5-6-sulfonic acid (FSA) concentration in the serum 4 h after gavage with FSA; results are shown normalized to control as 2 different experimental datasets were combined. Data are expressed as mean ± SEM. **b** *n* = 5; **c**–**g** *n* = 7–10; **h** *n* = 3–5; **i**–**l** *n* = 7–10. *p < 0.05, **p < 0.01, ***p < 0.001; one-way ANOVA, followed by Tukey’s multiple comparison test

We next assessed if the alterations induced by intestinal microbial depletion could be reversed following cessation of Abx treatment. We allowed spontaneous microbial recolonization for 21 days (Fig. 5a). To confirm bacterial recolonization, we assessed the bacterial load in the feces of the different groups. Bacterial load in the Abx-withdrawal (microbiota recovery) group was comparable to the control group (Figure S6). The Abx-withdrawal led to a slightly lower body weight as compared to the other two groups by the end of the experiment (Fig. 5b). Alterations in gut morphology induced by Abx treatment were normalized following bacteria recolonization (Fig. 5c, d); Abx-withdrawal mice had reduced cecal weight (Fig. 5c) and shorter small intestinal length compared to the Abx-treated group (Fig. 5d). No changes in colon length were observed (Fig. 5e). The fecal pellet output in response to stress also returned to baseline levels after recolonization (Fig. 5f), with a tendency (*p* = 0.10) to recovery in the wet:dry ratio of the feces (Fig. 5g).

To further explore the effects of bacterial depletion and recovery on secretion, segments of distal ileum were collected and mounted in Ussing chambers. No differences in ion transport, as measured by changes in short-circuit current (ΔIsc), were observed between Abx-treated and Abx-withdrawal groups when tissues were treated with veratridine, a stimulator of nerve-mediated secretion (Fig. 5h, left panel). However, when tissues were treated with carbachol, to directly activate the intestinal epithelium, we observed increased ΔIsc in tissues isolated from Abx-treated mice, which was not observed in the Abx-withdrawal group (Fig. 5h, right panel). These results suggest a role for a bacteria-dependent regulation of intestinal ion transport by epithelial cells.

The motility patterns evaluated by three different tests also demonstrated a recovery to initial baseline levels after bacterial recolonization (Fig. 5i–k). Lastly, evaluating intestinal permeability, we observed an elevated concentration of FSA in the serum of Abx-treated mice, and this returned to baseline levels in the Abx-withdrawal group (Fig. 5l). Here, we also investigated whether the distal ileum may be contributing to the increased gut permeability of the Abx-treated mice. We measured the transepithelial electrical resistance (TER) of the distal ileum in the Ussing chambers. Interestingly, no differences were observed among the three groups (control: 27.17 ± 1.46 Ω/cm^2^; Abx: 32.18 ± 7.19 Ω/cm^2^; Abx-withdrawal: 31.39 ± 3.01 Ω/cm^2^; one-way ANOVA). Overall, spontaneous reconstitution of the intestinal microbiota restored most of the altered physiological parameters induced by Abx treatment.

**Fig. 6:**
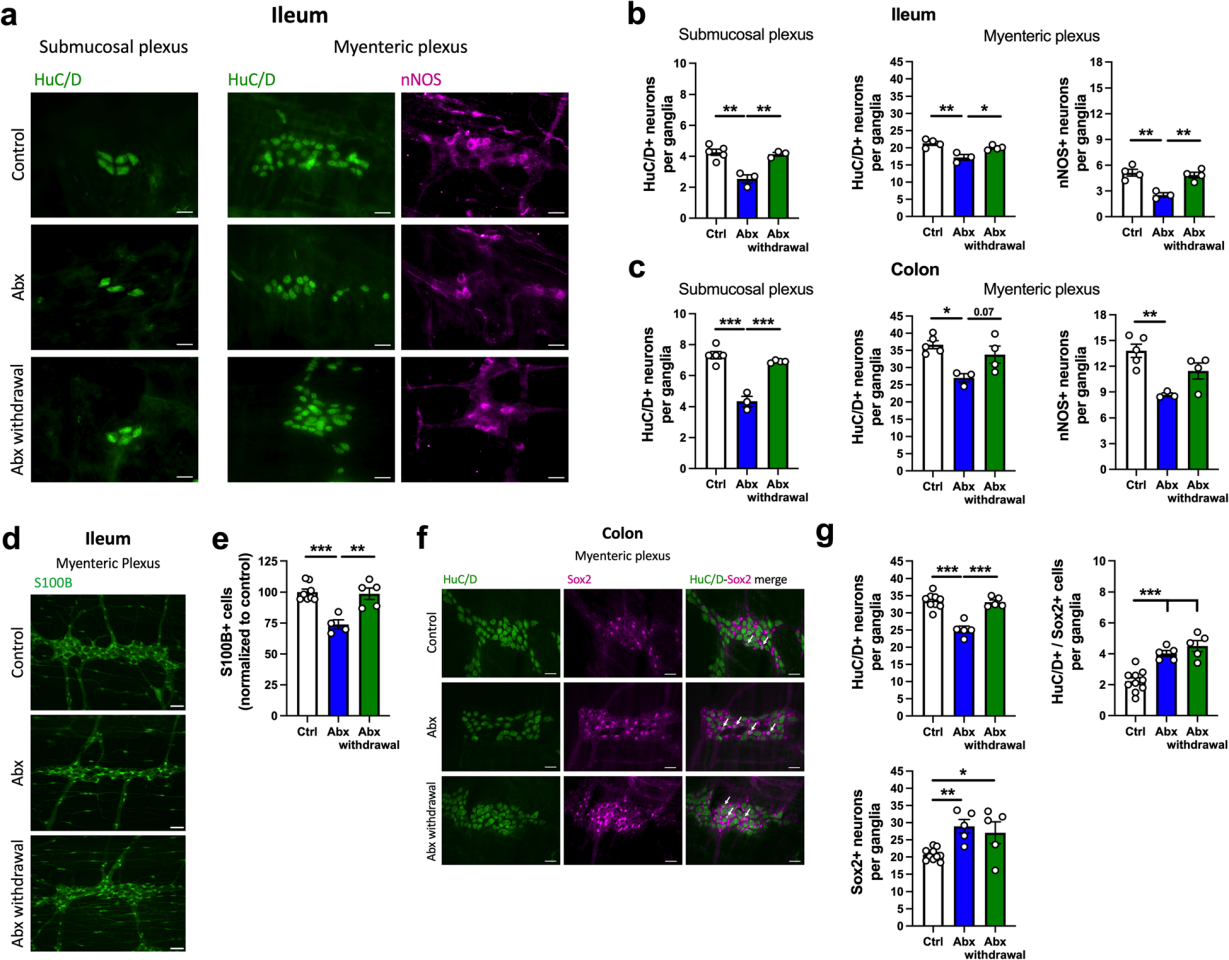
Neuronal and glial loss induced by antibiotic (Abx) treatment are reversed by spontaneous microbial recolonization and are accompanied by enteric neurogenesis. **a** Representative immunofluorescent images of ganglia in the submucosal and myenteric plexuses: HuC/D^+^ (green) and nNOS^+^ (magenta) neurons in the ileum. Scale bar: 30 μm. **b** Number of HuC/D^+^ neurons in the submucosal (left panel) and myenteric plexus (middle panel) in whole-mount preparations of the ileum. The number of nNOS^+^ neurons in the myenteric plexus (right panel) in the ileum. **c** Number of HuC/D^+^ neurons in the submucosal (left panel) and myenteric plexus (middle panel) in whole-mount preparations of the colon. The number of nNOS^+^ neurons in the myenteric plexus (right panel) in the colon. **d** Representative immunofluorescent images of ganglia in the ileal myenteric plexus showing S100B^+^ enteric glial cells (EGC, green). Scale bar: 50 μm. **e** Relative number of S100B^+^ EGC in the ileal myenteric plexus. **f** Immunofluorescence images of representative ganglia of the colonic myenteric plexus: HuC/D^+^ (green) and Sox2^+^ (magenta) cells. Arrows represent double-labelled HuC/D^+^/Sox2^+^ neurons. Scale bar: 30 μm. **g** Number of HuC/D^+^ neurons (upper left panel), Sox2^+^ cells (lower panel), and double-labelled HuC/D^+^/Sox2^+^ neurons (upper right panel) in the myenteric plexus of the proximal colon. Data are expressed as mean ± SEM unless stated otherwise. **a**–**c** *n* = 3-5; **d**–**g** *n* = 5–10. *p < 0.05, **p < 0.01, ***p < 0.001; one-way ANOVA, followed by Tukey’s multiple comparison test

Next, we sought to determine the effect of the spontaneous bacterial recolonization on neurons and EGC of the ENS (Fig. 6). Given the normalization of many of the alterations in GI physiology driven by Abx treatment (Fig. 5), and the evidence for increased enteric neurogenesis after Abx treatment (Fig. 4), we hypothesized that intestinal microbial recolonization would restore the neuronal population in the ENS. Indeed, we observed a recovery of HuC/D^+^ neurons in the submucosal plexus and a recovery of HuC/D^+^ and nNOS^+^ neurons in the myenteric plexus in the ileum of the Abx-withdrawal group (Fig. 6b). A similar tendency was observed in the colon regarding a restoration of HuC/D^+^ and nNOS^+^ neurons in the Abx-withdrawal group (Fig. 6c and Figure S7). The neuronal recovery was also accompanied by a complete normalization of the S100B^+^ EGC in the ileal myenteric plexus (Fig. 6d, e), illustrating the plasticity of the ENS. Moreover, we tested the modulation of neurogenesis after the microbiota recovery (Fig. 6f, g) and observed the restoration of HuC/D^+^ neurons as expected, but also a sustained increase in Sox2^+^ cells and HuC/D^+^/Sox2^+^ neurons in the Abx withdrawal group (Fig. 6g).

Overall, our data show that the presence of microbial communities in the gut is a major modulator of GI function and ENS integrity in the adult mouse, and that Abx-induced alterations in GI physiology and neuroanatomy of the ENS are largely reversible following spontaneous reconstitution of the microbiota.

### LPS supplementation prevents Abx-induced neuronal loss, but does not normalize GI function

**Fig. 7:**
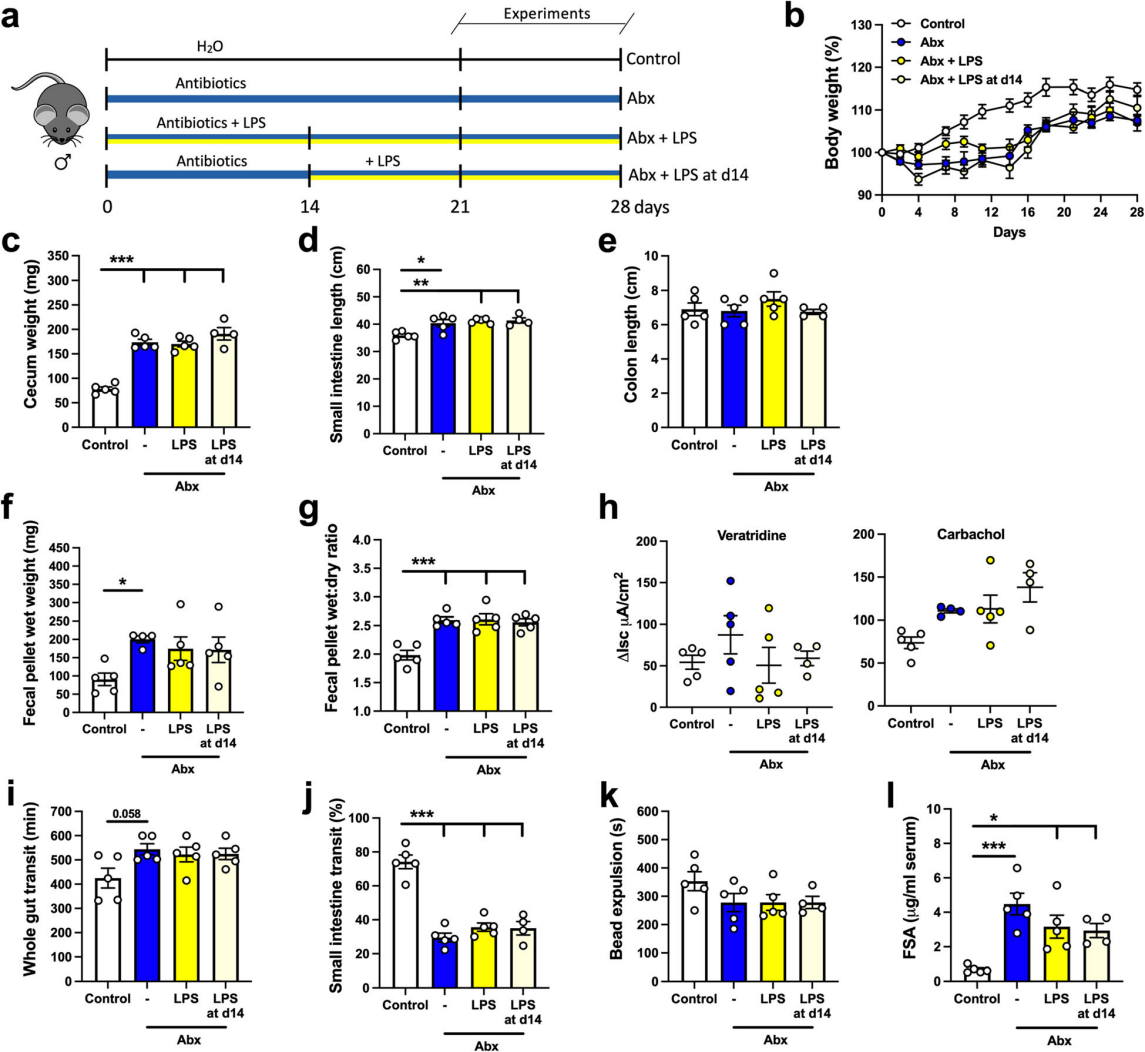
Supplementation with liposaccharide (LPS) does not normalize alterations in GI function induced by antibiotic (Abx) treatment. **a** Male mice treated with Abx were either supplemented with LPS (50 μg/ml) in the drinking water concomitant with Abx treatment (Abx + LPS) or after 14 days of Abx treatment (Abx + LPS at d14). Experiments were performed between 21-28 days after the beginning of the Abx treatment. **b** Body weight variation over the course of the experiment (two-way ANOVA). **c–e** Intestinal anatomical parameters; **c** cecal wet weight, **d** small intestinal length, and **e** colon length. **f** Fecal pellet wet weight and **g** wet:dry ratio of the feces measured after a 1-h novel environment stress. **h** Ion transport evaluated in ileal preparations mounted in Ussing chambers after stimulation with veratridine (10μM) or carbachol (100μM). **i** Whole gut transit time. **j** Small intestinal transit distance (as a % of total intestinal length) measured 15 min after gavage with dye. **k** Distal colonic motility measured by bead expulsion time. **l** Intestinal permeability assessed by fluorescein-5-6-sulfonic acid (FSA) concentration in the serum 4 h after gavage with FSA. Data are expressed as mean ± SEM. *n* = 4–5. *p < 0.05, **p < 0.01, ***p < 0.001; one-way ANOVA, followed by Tukey’s multiple comparison test

Next, we sought to investigate potential mechanisms by which the intestinal microbiota influences GI function and integrity of the ENS. LPS is a component of the cell wall of Gram-negative bacteria and is a known ligand of TLR4, a receptor previously described as important in the regulation of gut motility and ENS neuronal survival [18]. LPS levels are reduced after Abx treatment [18]. Hence, we sought to test whether supplementation with LPS (50 μg/mL) in the drinking water, at a concentration reported to modulate GI motility in mice [47], could attenuate or reverse the changes in GI function and ENS structure (Fig. 7a). Briefly, one group received LPS supplementation concomitant with the Abx treatment (LPS group), and a second received LPS supplementation after 14 days of Abx treatment (LPS at d14); a timepoint in which alterations caused by Abx treatment were fully established (Figs. 1, 2, and 3). No differences in body weight were detected among the different groups at the end of the experiment (Fig. 7b). Changes in gut morphology caused by Abx treatment, such as an enlarged cecum and longer small intestine, were not reversed by LPS supplementation (Fig. 7c–e). Functionally, LPS supplementation had no significant effects on fecal pellet wet weight after novel environment stress or the wet:dry ratio of the feces (Fig. 7f, g), nor did it normalize ΔIsc induced by veratridine or carbachol (Fig. 7h). As observed in previous experiments, Abx treatment slowed motility (Fig. 7i–k), which was not affected by LPS supplementation. Lastly, LPS supplementation had no effect on the alterations in intestinal permeability or TER induced by depletion of the intestinal microbiota (Fig. 6i; TER at d14: control: 27.14 ± 2.17 Ω/cm^2^; Abx: 29.36 ± 2.77 Ω/cm^2^; Abx + LPS: 32.65 ± 1.14 Ω/cm^2^; Abx + LPS: 35.85 ± 1.31 Ω/cm^2^; one-way ANOVA). Taken together, our LPS supplementation regimens (concomitant with Abx administration or after 14 days of Abx treatment), did not normalize the Abx-induced changes in GI structure or physiology.

**Fig. 8:**
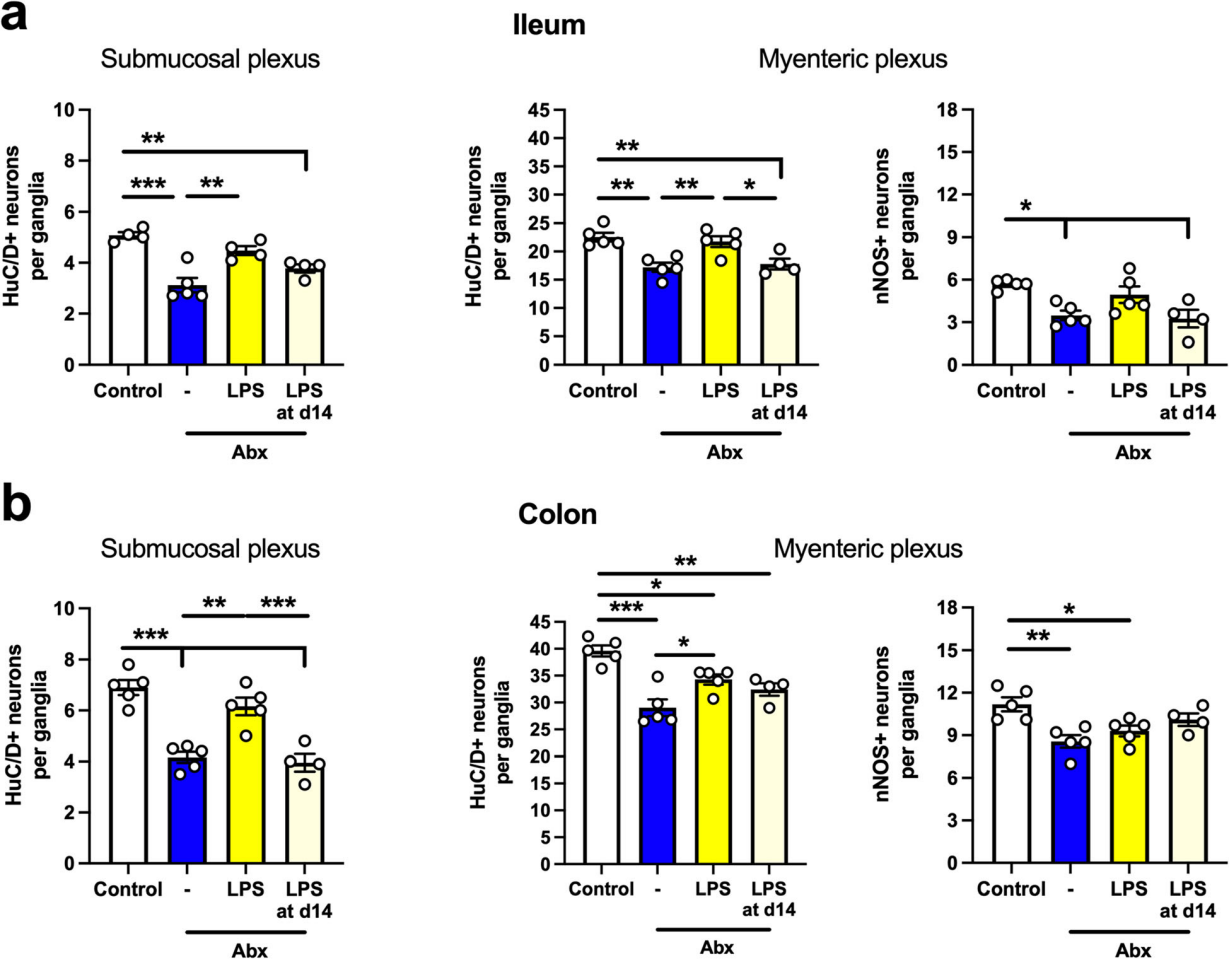
Lipopolysaccharide (LPS) supplementation prevents neuronal loss induced by antibiotic (Abx) treatment. **a** Number of HuC/D^+^ neurons in the submucosal (left panel) and myenteric plexus (middle panel) in whole-mount preparations of the ileum. Number of nNOS^+^ neurons in the myenteric plexus (right panel) in the ileum. **b** Number of HuC/D^+^ neurons in the submucosal (left panel) and myenteric plexus (middle panel) in whole-mount preparations of the colon. Number of nNOS^+^ neurons in the myenteric plexus (right panel) in the colon. Data are expressed as mean ± SEM. *N* = 4–5; 10 ganglia counted per mouse. *p < 0.05, **p < 0.01, ***p < 0.001; one-way ANOVA, followed by Tukey’s multiple comparison test

Although no significant changes in GI function were observed in Abx-treated mice supplemented with LPS, striking neuronal changes were observed (Fig. 8). In the ileum, Abx treatment reduced the number of HuC/D^+^ neurons in both plexuses (Fig. 8a, left and middle panels), as observed in our previous experiments. Interestingly, concomitant administration of LPS with Abx prevented the loss of HuC/D^+^ neurons (Fig. 8a, left and middle panels). However, beginning LPS supplementation after 14 days of Abx treatment was not able to reverse the Abx-induced neuronal loss (Fig. 8a, left and middle panel). Moreover, a parallel effect was seen on the nitrergic neuronal subpopulation; nNOS^+^ neurons were reduced in the Abx group compared to control, but this effect was attenuated with concomitant supplementation with LPS (Fig. 8a, right panel). Similar trends were observed in the colon (Fig. 8b). Both submucosal and myenteric plexuses had a reduction in the number of HuC/D^+^ neurons in the Abx-treated mice. This effect was attenuated in mice supplemented with LPS during Abx treatment, but not when LPS was given after 14 days of Abx treatment (Fig. 8b, left and middle panels). However, the number of nNOS^+^ neurons was not maintained by concomitant LPS supplementation in the colon (Fig. 8b, right panel). Together, these results suggest a role for LPS in neuronal survival, but not in ENS recovery (i.e., neurogenesis).

### SCFA supplementation rescues neuronal loss induced by Abx treatment, but has a minor effect on GI function

**Fig. 9:**
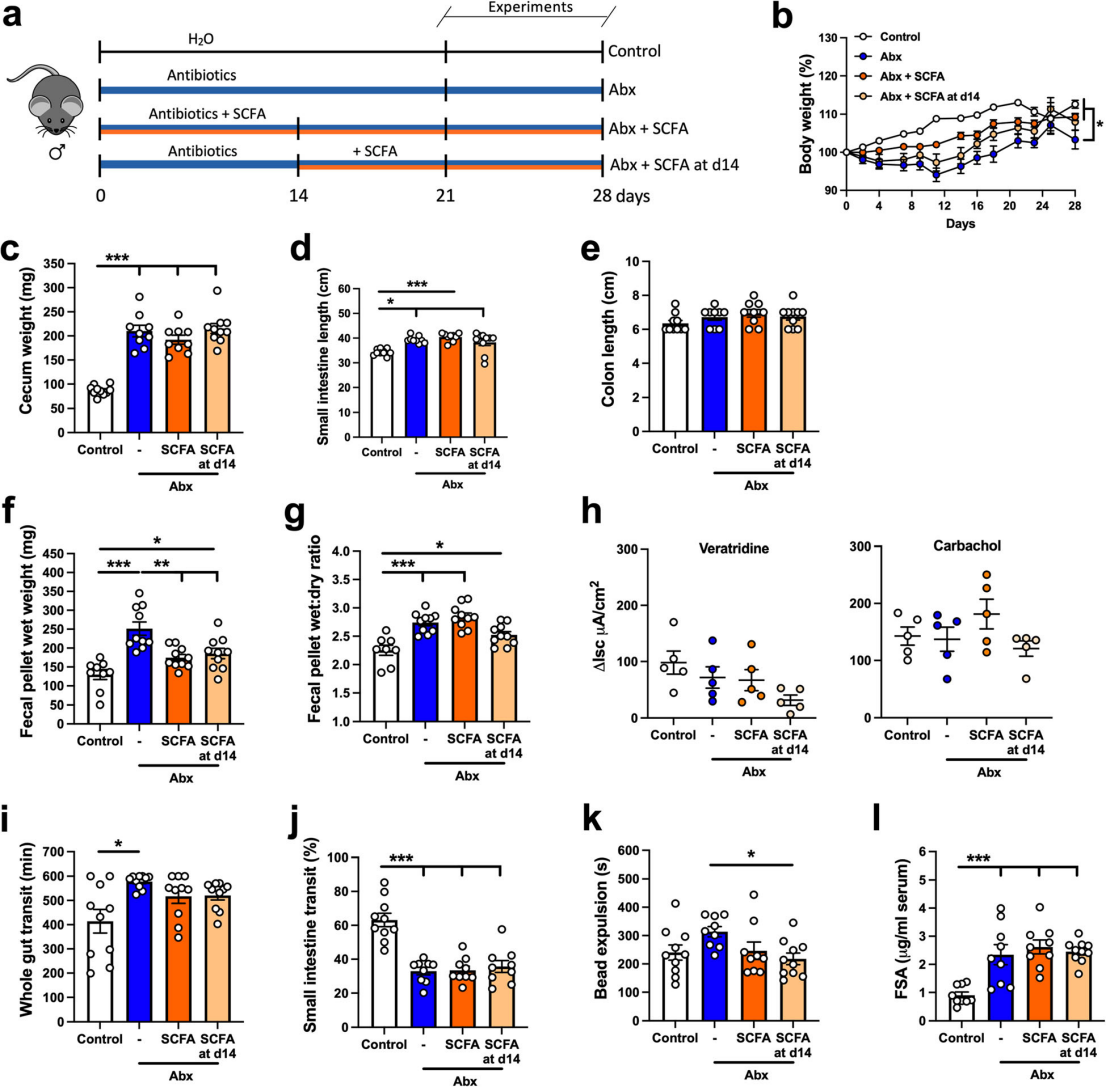
Supplementation with short-chain fatty acids (SCFA) does not restore altered motility or intestinal permeability induced by antibiotic (Abx) treatment but normalizes gut responses to stress. **a** Male mice treated with Abx were either supplemented with a mixture of SCFA (67.5 mM of acetate, 25.9 mM of propionate, 40 mM of butyrate) in the drinking water concomitant with Abx treatment (Abx + SCFA) or after 14 days of Abx treatment (Abx + SCFA at d14). Control and Abx-only (Abx) water were pH- and sodium-matched to the SCFA mixture. Experiments were performed between 21-28 days after the beginning of the Abx treatment. **b** Body weight variation over the course of the experiment (two-way ANOVA, followed by Tukey’s multiple comparison test). **c–e** Intestinal anatomical parameters; **c** cecal wet weight, **d** small intestinal length, and **e** colon length. **f** Fecal pellet wet weight and **g** wet:dry ratio of the feces measured after a 1-h novel environment stress. **h** Ion transport evaluated in ileal preparations mounted in Ussing chambers after stimulation with veratridine (10μM) or carbachol (100μM). **i** Whole gut transit time. **j** Small intestinal transit distance (as a % of total intestinal length) measured 15 min after gavage with dye. **k** Distal colonic motility measured by bead expulsion time. **l** Intestinal permeability assessed by fluorescein-5-6-sulfonic acid (FSA) concentration in the serum 4 h after gavage with FSA. Data are expressed as mean ± SEM. **b**–**g** *n* = 9–10; **h** *n* = 5; **i**–**l** *n* = 9–10. *p < 0.05, **p < 0.01, ***p < 0.001; one-way ANOVA, followed by Tukey’s multiple comparison test

Finally, we investigated the effects of SCFA supplementation in Abx-treated mice. The SCFA mix (67.5 mM of acetate, 25.9 mM of propionate, 40 mM of butyrate) [48] was administered in the drinking water concomitant with the Abx treatment (Abx + SCFA) or after 14 days of Abx treatment (Abx + SCFA at d14) (Fig. 9a).

A slightly reduced body weight was observed in the Abx group by the end of the experiment (Fig. 9b). Alterations in gut morphology caused by Abx treatment, such as enlarged cecum (Fig. 9c) and longer small intestine (Fig. 9d) were not affected by SCFA supplementation. No changes were observed in the colon length (Fig. 9e). SCFA supplementation, both concomitant and following 14 days of Abx treatment, reduced the elevated weight of fecal pellet output measured after the novel environment stress (Fig. 9f). The wet:dry ratio increase in the feces was not affected by SCFA treatment (Fig. 9g). No effects on ion transport in the ileum, nor motility or intestinal permeability, were observed in Abx-treated mice supplemented with SCFA (Fig. 9h–l; ileum TER at d14: control: 26.77 ± 1.38 Ω/cm^2^; Abx: 35.84 ± 3.91 Ω/cm^2^; Abx + SCFA: 33.25 ± 2.98 Ω/cm^2^; Abx + SCFA: 37.75 ± 2.49 Ω/cm^2^; one-way ANOVA).

**Fig. 10:**
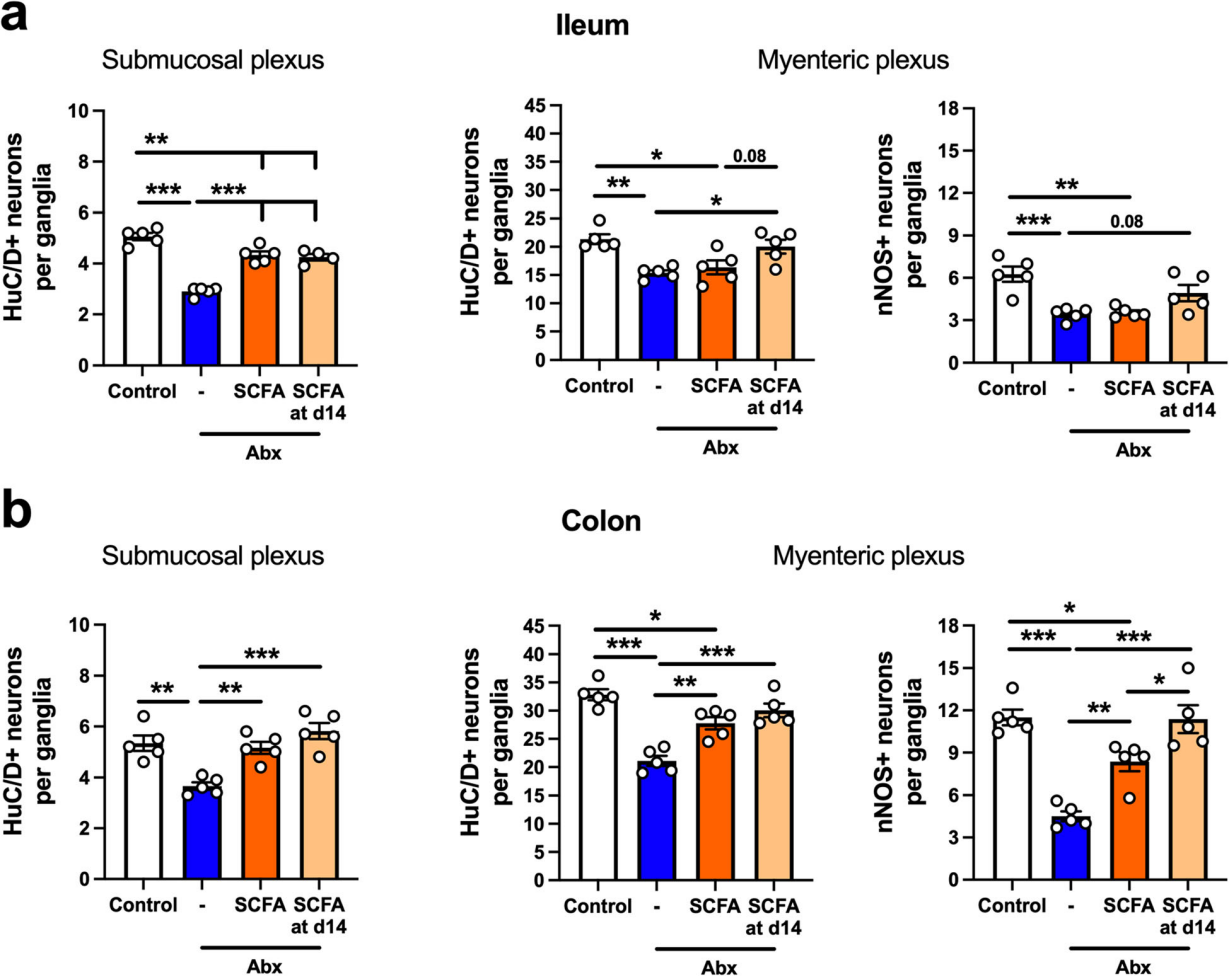
Short-chain fatty acids (SCFA) induce recovery of neuronal loss caused by antibiotic (Abx) treatment. **a** The number of HuC/D^+^ neurons in the submucosal (left panel) and myenteric plexus (middle panel) in whole-mount preparation of ileum. The number of nNOS^+^ neurons in the myenteric plexus (right panel) in the ileum. **b** Number of HuC/D^+^ neurons in the submucosal (left panel) and myenteric plexus (middle panel) in whole-mount preparations of the colon. Number of nNOS^+^ neurons in the myenteric plexus (right panel) in the colon. Data are expressed as mean ± SEM. *n* = 4–5; 10 ganglia counted per mouse. *p < 0.05, **p < 0.01, ***p < 0.001; one-way ANOVA, followed by Tukey’s multiple comparison test

Given the selective effects of SCFA supplementation on the functional parameters altered by Abx-induced intestinal microbiota depletion, we sought to determine if there was an effect on the neuroanatomical alterations in the ENS. Interestingly, a clear effect of the SCFA supplementation was observed in the submucosal plexus of both the ileum and the colon (Fig. 10a, b, left panels). A reduced number of HuC/D^+^ neurons was observed after Abx treatment, which was attenuated in both groups that received SCFA supplementation. In the ileal myenteric plexus, we observed neuronal recovery in mice supplemented with SCFA after 14 days of Abx treatment, but surprisingly, we did not observe a preservation of neurons when SCFA were delivered concomitant with Abx (Fig. 10a, middle panel). Additionally, no recovery effect was seen in the nitrergic nNOS^+^ neuronal subpopulation (Fig. 10a, right panel). In the colonic myenteric plexus, mice treated with SCFA at both timepoints had a higher number of HuC/D^+^ and nNOS^+^ neurons when compared to the Abx group (Fig. 10b, right panel). Due to this effect on neuronal recovery observed in the Abx + SCFA at the d14 group, we sought to evaluate whether SCFA would also regulate the number of S100B^+^ EGC in the ileal myenteric plexus, which was the only region where EGC were sensitive to Abx treatment (Fig. 3f). Here we observed a similar reduction in the S100B^+^ EGC numbers in the ileal myenteric plexus, however, no differences were observed after SCFA treatment (S100B^+^ EGC normalized to control: Control: 100 ± 1.9; Abx: 78.2 ± 4.1 (*p* < 0.05 compared to Control); Abx + SCFA: 87.2 ± 6.3 (*p* > 0.05 compared to Abx); Abx + SCFA at d14: 82.6 ± 5.0 (*p* > 0.05 compared to Abx); one-way ANOVA, followed by Tukey’s multiple comparison test). These results suggest a specific effect of SCFA on enteric neurons.

**Fig. 11:**
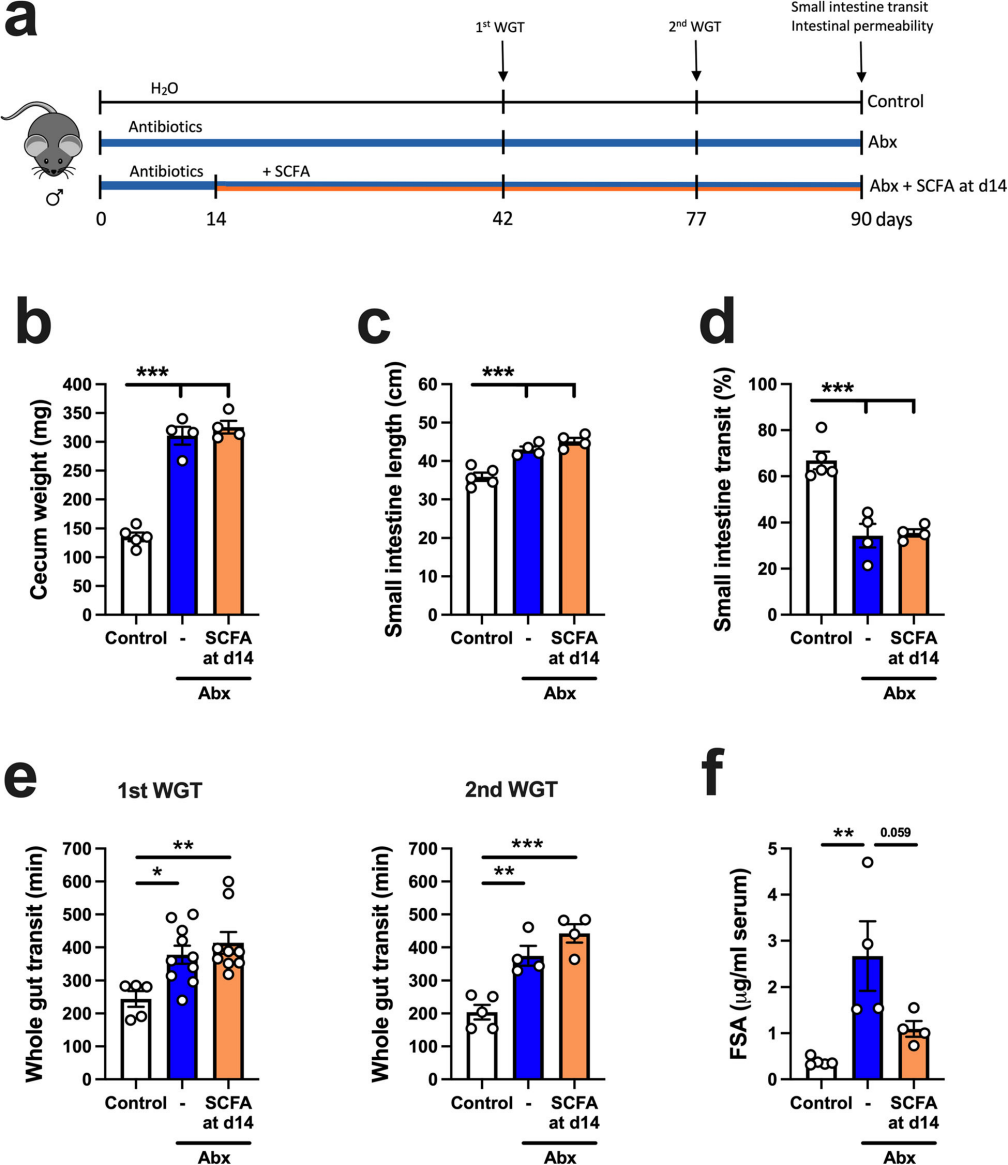
Short-chain fatty acid (SCFA) treatment does not restore antibiotic (Abx)-induced changes in motility, but improves intestinal barrier function. **a** Experimental design. Male mice treated with Abx were supplemented with a mixture of SCFA (67.5 mM of acetate, 25.9 mM of propionate, 40 mM of butyrate) in the drinking water after 14 days of Abx treatment (Abx + SCFA at d14). Control and Abx-only (Abx) water were pH- and sodium-matched to the SCFA mixture. Whole gut transit (WGT) test was performed on day 42 and day 77. Small intestinal transit test and intestinal permeability were assessed at 90 days. **b–c** Intestinal anatomical parameters; **b** cecal wet weight, **c** small intestinal length. **d** Small intestinal transit distance (as a % of total intestinal length) measured 15 min after gavage with dye. **e** Whole gut transit time tested on day 42 (1st WGT) and on day 77 (2nd WGT). **f** Intestinal permeability assessed by fluorescein-5-6-sulfonic acid (FSA) concentration in the serum 4 h after gavage with FSA. Data are expressed as mean ± SEM. *n* = 4–10. *p < 0.05, **p < 0.01, ***p < 0.001; one-way ANOVA, followed by Tukey’s multiple comparison test

Considering that SCFA restored the neuronal deficit induced by Abx treatment, but had only a minor effect on GI function, we asked whether a longer treatment regimen (90 days) with SCFA could restore the physiological alterations induced by Abx treatment (Fig. 11a). Morphological alterations induced by Abx treatment, such as increased cecum weight and longer small intestine length, were not restored by SCFA treatment (Fig. 11b, c). Moreover, long-term SCFA treatment did not restore the impaired motility induced by Abx treatment (Fig. 11d, e). We performed the whole gut transit test at two different timepoints, day 42 and day 77, and observed no difference between Abx and Abx+SCFA d14 groups (Fig. 11e). Similarly, no changes occurred after SCFA treatment when evaluating small intestine transit (Fig. 11d). Interestingly, SCFA restored the Abx-induced increase in intestinal permeability (Fig. 11e). These data suggest that SCFA may play a role in regulating barrier function, but their supplementation is insufficient to normalize most physiological functions perturbed by Abx treatment.

Together, we observed a role for SCFA in modulating enteric neurons in a region-specific manner, having the greatest effect in the submucosal plexuses and on colonic myenteric neurons.

## Discussion

In this study, we show the extent to which the intestinal microbiota regulates the structure and function of the GI tract in the adult mouse. We observed significant effects on small intestinal length, cecal mass, GI motility, ion transport, and intestinal permeability that were independent of the sex of the mice. Abx-treated mice displayed slower gut transit time, alterations in ion transport, increased gut permeability, and enhanced fecal output in a novel environment stress paradigm. These alterations were accompanied by neuroanatomical changes in the ENS, including an indiscriminate loss of enteric neurons and region-specific loss of EGC. Spontaneous recovery of the microbiota restored the functional and morphological deficits, including motility and permeability alterations. Remarkably, spontaneous recolonization was also associated with the recovery of neurons, through enteric neurogenesis. Investigating which components of the microbiota were responsible for these effects, we found that neither LPS nor SCFA restored Abx-induced changes in gut function, though SCFA supplementation was able to reverse the stress-induced enhancement of fecal output. Despite not reversing most of the functional deficits, LPS was shown to enhance neuronal survival, and SCFA enhanced neuronal survival and promoted enteric neurogenesis. Why these significant changes in the structure of the ENS are not accompanied by functional recovery remains to be determined. Nevertheless, these findings suggest that other microbial components are critical for initiating the physiological function of new enteric neurons.

Reports in both germ-free and Abx-treated rodents have described alterations in baseline GI function, suggesting a role for the microbiota in the regulation of motility, ion transport, and permeability [21, 23, 24, 26, 49–51]. While sex-dependent differences in the composition of the microbiota have been described [52], we observed similar physiological alterations in both sexes when bacteria were depleted by Abx treatment (e.g., dysmotility and increased gut permeability). These data suggest that, even though initial bacterial composition may be different, a core signaling mechanism is present in both microbiota, which, upon depletion, led to similar alterations in GI function in both sexes of mice. While previous work has shown that Abx exposure can directly influence neuronal function and intestinal motility patterns in vitro [53], others have described similar effects to intestinal physiology when analyzing both germ-free and Abx-treated mice, suggesting that the alterations in GI function are more likely to be related to the absence or depletion of the intestinal microbiota, rather than a side effect of the Abx treatment [18, 25, 54]. Additionally, to address whether the functional changes induced by depletion of the microbiota were permanent, we allowed mice to recover for 21 days after 14 days of Abx treatment (Fig. 5). Suez and colleagues reported that in mice, full restoration of the microbiota via spontaneous recolonization after Abx treatment can take longer than 21 days [55]. Nevertheless, we observed a significant normalization of GI function and overall microbial numbers following the 21-day recovery period. In agreement with our findings, germ-free and Abx-treated mice recolonized over a shorter period or colonized with reduced bacterial diversity (e.g., altered Schaedler flora), recovered specific GI functions, such as motility and intestinal permeability [15, 25, 26]. This suggests that the mechanism(s) driving these processes are likely not dependent on specific bacteria, but rather on common bacterial signaling pathways, such as those related to the MAMPs or abundant microbial secreted molecules. Therefore, we sought to test whether supplementation with either LPS or SCFA, both common microbe-derived mediators, could reverse or attenuate the changes induced by Abx treatment. These molecules have been associated with modulation of many different properties of the GI tract, including physiological (e.g., motility and intestinal permeability) and immune processes [34, 35, 47, 56, 57], and both have reduced concentrations after depletion of the microbiota [18, 58]. Surprisingly, few beneficial effects on GI function were observed when Abx-treated mice were supplemented with either LPS or SCFA, with the only parameters normalized being the gut responses to a stress stimulus and the intestinal permeability. We showed that pellet output number after novel environment stress was recovered by SCFA supplementation, suggesting a potential role for SCFA in the modulation of the local response to stress, and long-term SCFA treatment restored the intestinal barrier. Thus, our results suggest a minor functional effect of LPS or SCFA supplementation, at least at the levels and intervals used in this study. This supports the notion of a more complex interplay between the host and the intestinal microbiota, likely involving multiple integrating mechanism(s) on the host to regulate GI physiology.

The ENS plays a fundamental role in regulating gut motility, secretion, and permeability [1, 59]. Previous studies have shown a modification of the ENS structure and function in germ-free rodents. These alterations consist of a reduction in neuronal density, including nitrergic neurons, and reduction in EGC, in addition to altered electrophysiological responses in enteric neurons [15, 18, 22, 54, 60]. Our results support the hypothesis that depletion of bacteria induces enteric neuronal loss. However, alterations in EGC were not as widespread as the changes observed in neuronal populations. Previous reports have shown a reduction in EGC bodies in the ileal mucosa, but no clear alterations in the myenteric plexus [22], and reduction in the density of glial processes in the colonic myenteric plexus, but no changes in the number of EGC [61]. In contrast, we observed no changes in the number of PLP1^+^ EGC cell bodies in the mucosa after Abx treatment in both ileum and colon (Fig. 3), and we showed a reduction in both the number of EGC and the density of glial processes specifically in the ileal myenteric plexus. Exactly why there are differences between our results and the published literature is not clear and requires further investigation.

Interestingly, upon recolonization, ENS neuronal and glial densities were restored in both the ileum and the colon. These data suggest a correlation between a restored ENS structure with the recovery of function (Fig. 5). Moreover, enteric neuronal recovery has been recently demonstrated by others using a similar protocol of post-Abx treatment recolonization [25], in which the authors suggested a role for enteric postnatal neurogenesis mediated by the microbiota. Our findings are entirely consistent with these observations and extend them showing that not only do nestin-positive progenitor cells increase [25], but also Sox2^+^ cells that potentially arise from glial progenitor cells [44, 46, 62]. Enteric postnatal neurogenesis refers to the production of new neurons in the GI tract from birth to adulthood. The concept of neurogenesis in the brain of mammals in specific regions has now been widely accepted [63], however, this process within enteric neurons has been largely studied in vitro [62, 64, 65]. Only recently has enteric neurogenesis been demonstrated in an in vivo setting, with either healthy animals [43] or under conditions of inflammation or injury [44, 62, 64, 66]. It has become clear that the cell source for postnatal enteric neurogenesis is a glial cell or glial cell subtype [62, 64, 67]. The two glial markers most commonly used to demonstrate the glial-to-neuronal production of new neurons have been nestin [25, 43, 65, 68] and Sox2 [44, 46]. Interestingly, these studies have focused on the myenteric plexus and, to date, no one has compared the submucosal and myenteric plexus in the same animals. Surprisingly, we observed differences in Sox2 expression mediated by microbiota depletion when comparing the response to bacterial depletion in the two plexuses. These findings suggest that different mechanisms govern neurogenesis in the two plexuses in adult animals and raise many questions on how enteric neurogenesis is regulated and the extent to which the intestinal microbiota contributes to these processes. Nevertheless, the importance of this mechanism is underscored by the recent work from Matheis and colleagues, in which the authors showed that infection-induced neuronal loss can be reversed following reconstitution of the normal microbiota after Abx treatment [69].

One of the potential mechanisms by which the intestinal microbiota may modulate the ENS is via TLRs and their MAMP ligands [70, 71]. The expression of different TLRs in the ENS has been demonstrated previously [32, 33]. Anitha et al. elegantly showed the importance of neuronal TLR4 signaling in the control of gut motility [18], which was further confirmed by others [72]. Additionally, it has been shown that low-dose LPS has neuroprotective effects in both the central nervous system and ENS in vitro [18, 73]. In our experiments, while low-dose LPS supplementation did not reverse or attenuate the alterations in GI function induced by the Abx treatment, we observed a beneficial effect on neuronal survival when LPS was administered concomitantly with the Abx treatment. Our results complement previous in vitro reports [18, 73], demonstrating a role for the LPS-TLR4 signaling pathway in enteric neuronal survival. While LPS supplementation prevented Abx-induced neuronal loss, it is important to note that later supplementation with LPS after 14 days of Abx treatment did not rescue the neuronal loss. In vitro analysis has shown that LPS stimulates enteric neural progenitor cell proliferation, however, it slowed cell differentiation [74]. Thus, LPS-TLR4 signaling is likely associated with neuronal survival, rather than cell differentiation/neurogenesis. In addition to TLR4, TLR2 has been identified as a key regulator of the ENS and gut motility [19, 32]. A recent report described the effect of TLR2 in Abx-induced neuronal loss and altered gut functions [25]. The authors were able to reverse the Abx-induced neuronal loss by administering lipoteichoic acid (LTA), a TLR2 agonist, suggesting a role for TLR2 activation in neurogenesis after Abx-induced neuronal loss [25]. In hippocampal neurons, a dynamic complex modulation of both TLR2 and TLR4 has been implicated in neuronal survival and differentiation/neurogenesis [75]. TLR2 deficiency impairs the differentiation of neural progenitor cells into mature neurons, highlighting its importance in the neurogenesis process. Additionally, TLR4 seems to counteract and regulate TLR2 function in hippocampal neurons [75]. Based on our results, and recent literature, we speculate that in the ENS, TLR4 signaling is important for neuronal survival, while TLR2 activation may be required for neurogenesis. Nevertheless, additional studies are needed to delineate the complex interplay between TLR2 and TLR4 and their contribution to ENS neuronal survival and neurogenesis.

In addition to MAMPs, it has been shown that microbial-derived metabolites can modulate ENS and GI function. Reports have described a role for SCFA in increasing the expression of tryptophan hydroxylase 1 (Tph1; i.e., rate-limiting enzyme in the 5-HT synthesis pathway in enterochromaffin cells) in vitro [36]. Interestingly, dysmotility has been correlated with reductions in 5-HT concentration in the gut in a reversible manner [20, 24, 36]. Furthermore, 5-HT has been associated with neurogenesis in the ENS, potentially via the 5-HT_4_ receptor [45, 61]. Thus, we sought to test the effects of SCFA supplementation on GI function and ENS structure. While SCFA supplementation had little effect on the alterations in GI function induced by microbial depletion, we observed that this regimen led to a recovery of the enteric neurons after Abx treatment. These data suggest a role for SCFA in neuronal survival and/or neurogenesis after neuronal loss. We postulate that a potential mechanism by which SCFA may be acting on neurogenesis is via the 5-HT_4_ receptor. Since SCFA increase Tph1 expression and levels of 5-HT in the gut [24, 36], and the 5-HT_4_ receptor has been implicated in neurogenesis [45, 61, 76], it is plausible to hypothesize a link between the two. On the other hand, expression of free-fatty acid receptors, of which SCFA are agonists, has been demonstrated in enteric neurons [77]; however, their role in neuronal survival/proliferation/differentiation is still unknown. Thus, additional experiments are needed to determine the discrete mechanism(s) responsible for these beneficial effects of SCFA supplementation in our studies.

The implications of bacterial depletion in adult human health are still under investigation, but changes in barrier function appear to be critical. Here we show that a transient increase in gut permeability after Abx treatment can be normalized following bacterial recolonization in mice. However, we have not identified which region of the gut is responsible for this increase in permeability. Other reports have described the importance of the microbiota in regulating gut mucosal function in germ-free mice ex vivo, including secretion and maturation of the epithelial barrier [51, 78]. In accordance with our results, similar outcomes were described in Abx-treated adult mice, in which a transient increase in gut permeability was observed in vivo [26]. We assessed the role of LPS and SCFA in the modulation of the gut barrier after microbiota disruption. Previously, both molecules have been demonstrated to influence gut permeability [57, 79]. Even though no recovery effect was observed after intervention with LPS in our Abx-treated mice, long-term treatment with SCFA was able to reduce intestinal permeability. Interestingly, the presence of metronidazole in an Abx mix, or when administered alone, is associated with increased gut permeability, likely playing a crucial role in the effects observed [26, 80]. Thus, future analysis of the bacteria being depleted or blooming after metronidazole treatment may shed light on potential microbe-driven mechanisms regulating intestinal permeability.

## Conclusions

Our results suggest a role for the gut microbiota in constantly regulating a variety of GI functions in adulthood, independent of sex. Depletion of gut bacteria altered GI motility, secretion, and permeability. Moreover, the microbiota is essential for the maintenance of the ENS integrity, likely regulating enteric neuronal survival and promoting neurogenesis. MAMPs, such as LPS, may regulate enteric neuronal survival, while SCFA treatment was found to be associated with both survival and neurogenesis. Thus, we provide additional understanding of host-microbe interactions that contribute to the regulation of GI structure and function and the organization of the ENS.

## Methods

### Animals

Male and female C57Bl/6 mice (Jackson Laboratories, Bar Harbor, ME, USA), 8–12 weeks old, were housed (*n* = 5/cage) in the animal facility at the University of Calgary at 22 ± 2 °C on a 12-h light–dark cycle. Mice had free access to sterilized food and water. Upon arrival, mice were randomized, followed by a 1-week acclimation period before the start of any experiment. PLP1-eGFP mice were developed by Dr. Wendy Macklin [40] and were bred in-house at the University of Colorado’s animal facility. Male and female PLP1-eGFP littermate mice, 8–12 weeks old, were used in the experiments. Mice were kept under the same conditions as described above. All animal procedures were approved by the University of Calgary’s Health Sciences Animal Care Committee (#AC19-0124) and the University of Colorado’s Anschutz Institutional Animal Care and Use Committee (#00088), in accordance with the guidelines established by the Canadian Council on Animal Care and the PHS Policy on Humane Care and Use of Laboratory Animals, respectively.

### Treatments

#### Broad-spectrum antibiotic treatment

Abx treatment was administered according to previously published protocols [54, 56]. Broad-spectrum Abx mix was diluted in sterilized water and consisted of ampicillin (1 g/L, A9518; Sigma-Aldrich, St. Louis, MO, USA), neomycin (1 g/L, N1876; Sigma-Aldrich), vancomycin (0.5 g/L, 94747; Sigma-Aldrich), and metronidazole (1 g/L, M3761; Sigma-Aldrich). Mice received the Abx solution for at least 14 days. Due to the body weight loss observed at the beginning of the Abx treatment in a pilot experiment (Figure S8), also shown by others [81], we opted to gradually introduce metronidazole in the Abx solution as previously described [38]. Briefly, no metronidazole was added on day 0; 0.25 g/L (25% of final concentration) was added at day 2; 0.50 g/L (50% of final concentration) was added at day 6; and 1.0 g/L (full concentration) was added at day 9. Abx solution was refilled every week. Body weight was assessed three times a week to monitor body weight changes. To assess the effects of spontaneous microbiota recolonization, bottles of Abx were swapped for regular autoclaved water at day 14. Mice had access to water for 21 days after the bottle swap to allow for microbiota recolonization.

#### Low-dose LPS supplementation

Low-dose LPS supplementation was administered as described previously [38, 56]. LPS (*Escherichia coli* O111:B4; L2630, Sigma-Aldrich) was diluted (50 μg/ml) in the Abx solution. Animals had free access to the Abx solution containing LPS from day 0, or after 14 days of Abx-treatment only, depending on the treatment group. Mice received LPS supplementation for 21 or 7 days prior to any experiment. LPS concentration was chosen based on promising beneficial effects on gut motility [47].

#### SCFA mix supplementation

A mixture of SCFA was administered according to previous reports [48, 82, 83]. SCFA mixture was composed of 67.5 mM of acetate (S2889, Sigma-Aldrich), 25.9 mM of propionate (P1880, Sigma-Aldrich), and 40 mM of butyrate (303410, Sigma-Aldrich). The mixture was diluted in the Abx solution. Solutions with no SCFA mixture were sodium matched. All solutions were pH controlled (7.4–7.6) and changed weekly.

### In vivo measurements

#### Novel environment stress response and fecal water content

Mice were placed individually in new bedding-free cages and monitored for 1 h. Fecal pellets were collected and weighed every 15 min. Feces were dried at 50 °C for 24 h and re-weighed. Wet and dry weights were used to generate the fecal pellet wet:dry ratio.

#### Whole gut transit time

The assessment of the whole gut transit time was performed immediately following the novel environment stress test. Mice were gavaged with 200 μL of the non-absorbable dye Evans Blue (5% suspended in 5% Gum Arabic; Sigma-Aldrich) and the period from the time of the gavage to the appearance of the first blue-colored fecal pellet was considered the whole gut transit time. Tests were normally ended at 480 min. However, if 20% or more of the animals being tested had not expelled a blue pellet by 480 min, they were allowed an extra 120 min of testing, with the test ending at 600 min.

#### Assessment of small intestine transit

Mice were gavaged with 200 μL of the non-absorbable dye Evans Blue (5% suspended in 5% Gum Arabic), and 15 min later, they were euthanized under isofluorane anesthesia. The small intestine was immediately removed and measured (pyloric sphincter to ileal–cecal junction). Distance traveled by the dye was also measured. Results are expressed as a percentage of the length covered by the dye over the total small intestinal length. This test was performed concomitantly with the intestinal permeability assay such that mice received the Evans Blue gavage 3 h 45 min following the FSA gavage for the intestinal permeability assay (see below).

#### Distal colonic transit time

We used the bead expulsion test to assess distal colonic propulsion. Mice were lightly anesthetized with isoflurane and a 2.5-mm spherical plastic bead coated in nail varnish was gently inserted 2 cm into the distal colon using a silicone pusher. Mice were placed in individual, bedding-free cages, and latency time for bead expulsion was recorded. After bead expulsion, mice were returned to their home cage. Experiments were repeated 3 times with 100 min intervals. The mean of 3 experiments was considered the time for bead expulsion.

#### Intestinal permeability assay

Mice were gavaged with 100 μL of 50 mg/ml FSA (478 daltons; Setareh Biotech, Eugene, OR, USA). After 4 h, mice were anesthetized under isofluorane. Blood was drawn by cardiac puncture and collected in a Microtainer SST Tube (BD Company, Franklin Lakes, NJ, USA) after which mice were euthanized and the small intestine removed to determine small intestinal transit as above. At room temperature, blood was allowed to clot for at least 30 min, followed by centrifugation at 2000 × *g* for 10 min. The supernatant (serum) was collected, pipetted (50 μL) in triplicate into a 96-well plate, and read at 485/535 nm in a spectrophotometer. Sample FSA concentration (μg/mL of serum) was determined using a standard curve.

### Tissue harvesting

After in vivo experiments, mice were either anesthetized with isoflurane and euthanized by cervical dislocation, or euthanized by CO_2_ asphyxiation followed by cervical dislocation. The entire gut was removed, and different regions were allocated for different analyses. The cecal contents were removed and the cecum was weighed. The length of the small intestine and colon was measured. The distal ileum was divided for immunofluorescence and Ussing chamber tests. The proximal colon was harvested for immunofluorescence.

### Immunofluorescence labeling

Dissection of submucosal and myenteric plexuses was done similarly in both the ileum and colon. To obtain whole-mount preparations, samples were first bathed in a solution containing 1 μM nifedipine (Sigma-Aldrich) diluted in PBS for 10 min. Next, tissues were opened along the mesenteric border and pinned out in a petri dish containing Sylgard coating with the serosal side facing down. Samples were fixed with either Zamboni’s fixative for 24 h at 4 °C, or specific for ChAT staining and PLP1-eGFP mice, with 4% paraformaldehyde (ThermoFisher Scientific, Waltham, MA, USA) for 2 h at 4 °C. Tissues were then washed with PBS containing 5% sodium azide (3 times 10 min each) and stored at 4 °C until further processing. To obtain submucosal and myenteric preparations, samples were dissected under a microscope; submucosal preparations were obtained by gently scraping the mucosa away and peeling off the submucosal plexus, and the myenteric preparations were acquired by stripping off the mucosa/submucosa layers and the circular muscle leaving a preparation consisting of the longitudinal muscle and associated myenteric plexus. Following the acquisition of submucosal and myenteric preparations, samples were processed for immunohistochemistry. First, samples were incubated with primary antibody (Table 1) for 48 h at 4 °C. The primary antibody solution was removed, samples were washed (3 times 5 min each) with PBS containing 0.1% Triton X-100 (Sigma-Aldrich), and incubated with secondary antibody (Table 1) for 1–2 h at room temperature followed by washing (3 times 5 min each) with PBS. When double labeling, samples were washed with PBS (3 times of 10 min each), and the second labeling was performed following the same protocol. Particularly when using anti-Sox2 antibodies, samples were pre-treated with DMSO (VWR International, Edmonton, AB, Canada) for 30 min at room temperature prior to primary antibody incubation. Whole-mount preparations were mounted with bicarbonate-buffered glycerol on microscope slides, stored at 4 °C under dark conditions for further analyses. All antibodies used were diluted in a PBS solution containing 0.1% Triton X-100, 10% bovine serum albumin (BSA), 5% sodium azide, 4% EDTA. Tissues from PLP1-eGFP mice were counterstained with DAPI (Invitrogen-ThermoFisher Scientific, Waltham, MA, USA) and mounted with Prolong Gold (Invitrogen-ThermoFisher Scientific).

**Table 1:** Antibody reagent list

*Antibodies*	*Dilution*	*Source*	*Identifier*
Mouse anti-HuC/D	1:200	Invitrogen	Cat#: A21271; RRID: AB_221448
Donkey anti-mouse Alexa Fluor 488	1:200	Jackson ImmunoResearch	Cat#: 715-546-151; RRID: AB_2340850
Sheep anti-nNOS	1:200	Millipore Sigma	Cat#: AB1529; RRID: AB_90743
Donkey anti-sheep Cy3	1:100	Jackson ImmunoResearch	Cat#: 713-165-147; RRID: AB_2315778
Goat anti-ChAT	1:50	Millipore Sigma	Cat#: AB144P; RRID: AB_90661
Donkey anti-goat Cy3	1:200	Jackson ImmunoResearch	Cat#: 705-165-147; RRID: AB_2307351
Rabbit anti-S100B	1:500	Agilent	Cat#: GA50461-2; RRID: AB_10013383
Donkey anti-rabbit Cy3	1:100	Jackson ImmunoResearch	Cat#: 711-165-152; RRID: AB_2307443
Goat anti-Sox2	1:200	R&D Systems	Cat#: AF2018; RRID: AB_355110
Donkey anti-goat Cy3	1:200	Jackson ImmunoResearch	Cat#: 705-165-147; RRID: AB_2307351
Rabbit anti-S100	1:200	ThermoFisher Scientific	Cat#: RB-044-AO; RRID: AB_60518
Donkey anti-rabbit Alexa Fluor 647	1:400	Invitrogen	Cat#: A-31573; RRID: AB_2536183
Mouse anti-Tuj1	1:200	Biolegend	Cat#: 801201; RRID: AB_ 2313773
Donkey anti-mouse Alexa Fluor 488	1:200	Jackson ImmunoResearch	Cat#: 715-546-151; RRID: AB_2340850
Goat anti-calretinin	1:200	Swant	Cat#: CG1; RRID: AB_10000342
Donkey anti-goat Cy3	1:200	Jackson ImmunoResearch	Cat#: 705-165-147; RRID: AB_2307351
Rat anti-Ki67	1:400	Invitrogen	Cat#: 14-5698-82; RRID: AB_10854564
Goat anti-rat Alexa Fluor 647	1:400	Invitrogen	Cat#: A21247; RRID: AB_141778
Human anti-Hu	1:10,000	Mayo Clinic	Cat#: N/A; RRID: AB_2314657
Goat anti-human Alexa Fluor 647	1:400	Invitrogen	Cat#: A21445; RRID: AB_2535862

To obtain cryosections from PLP1-eGFP mice, distal ileum and colon were harvested and fixed in 4% paraformaldehyde (ThermoFisher Scientific) diluted in PBS for 24 h at 4 °C. After rinsing with PBS, tissues were submerged in 20% sucrose in PBS for 24 h at 4 °C and embedded in O.C.T. compound (ThermoFisher Scientific) on a bed of dry ice. Cryosections were cut at 20 μm and collected on poly-d-lysine (Sigma-Aldrich) coated slides, with intervals of 40–80 μm between each collected section. Cryosections were equilibrated to room temperature, fixed in 4% paraformaldehyde for 20 min, and blocked in 10% donkey serum, 10% BSA and 1% Triton X-100 diluted in PBS for 1 h at room temperature. Here, a rabbit polyclonal antibody for S100 (ThermoFisher Scientific) at 1:200 diluted in blocking buffer was applied to the sections overnight at 4 °C. Sections were washed with PBS and incubated in donkey anti-rabbit secondary antibody conjugated to Alexa Fluor-647 (Invitrogen) at 1:400 diluted in blocking buffer for 1 h at room temperature. For Ki67 immunolabeling, a similar protocol was used, applying Ki67 monoclonal antibody (ThermoFisher Scientific) and HuC/D (Mayo Clinic). Sections were washed with PBS, counterstained with DAPI (Invitrogen), and coverslipped with Prolong Gold (Invitrogen).

### Quantification of enteric neurons and EGC

Analyses for neuronal (HuC/D^+^, nNOS^+^, ChAT^+^, Calretinin^+^, Sox2^+^) counts and S100B^+^ EGC were done using a Zeiss Axioplan fluorescence microscope (Zeiss Canada, Toronto, ON, Canada). Analyses of PLP1-eGFP mouse samples were undertaken on an Olympus IX83 motorized inverted microscope (Olympus, Center Valley, PA, USA). For neuronal counts, 10 ganglia were randomly selected and immunolabelled cells were quantified, within each preparation, either submucosal or myenteric. Data for each animal is composed of the mean of the 10 ganglia counted. For Tuj1^+^ neuronal fiber analysis, the first images were captured using the Zeiss Axioplan fluorescence microscope (Zeiss Canada) applying the same exposure time to all samples. By using the FIJI software (ImageJ, NIH, Bethesda, MD, USA) for image analysis, the ganglia were delineated, and the fluorescence intensity measured. Background fluorescence intensity was subtracted to get a corrected fluorescence intensity. Ten ganglia were measured per region, and averaged for the final value. For S100B^+^ EGC numbers, a total ganglionic area of 0.020–0.036 μm^2^ for submucosal and 0.20–0.23 μm^2^ for myenteric was analyzed per mouse, and S100B^+^ cells were counted within this area. Data are presented as normalized to control values due to variation in tissue stretching during fixation among cohorts. Mucosal PLP1^+^ EGC were manually quantified in villi and crypts along the entire circumference of the ileum. An average of 35 intact villus-crypt units were counted per mouse. For colonic analysis, an average of 1.5 mm^2^ of mucosal area was analyzed per mouse. Percent area measurements of the PLP1^+^ signal in images of whole-mount preparations were analyzed with FIJI (ImageJ, NIH) to determine glial cell density. An average of 4 images were analyzed per animal. All quantification was done by an experimenter blinded to the treatments.

### In vivo cell proliferation assay

Mice received 100 μL of 10 mg/mL of EdU (ThermoFisher Scientific) diluted in molecular grade water by intraperitoneal injection 7 days following the start of Abx treatment or 1 mg of EdU per mouse. Click-IT Plus EdU Alexa Fluor 594 Imaging Kit (Invitrogen) was used according to manufacturer instructions to visualize the EdU in cryosections of the distal ileum and colon of the PLP1-eGFP mouse.

### Measurement of ion transport and transepithelial electrical resistance

Immediately after euthanasia, full-thickness segments of terminal ileum were removed, opened along the mesenteric border, cleaned of luminal contents, and mounted in Ussing chambers (Physiologic Instruments, San Diego, CA, USA) with an exposed area of 0.3 cm^2^. The tissues were bathed at 37 °C in oxygenated (95% O_2_–5% CO_2_) Krebs solution (pH 7.4) with 10 mM of glucose and mannitol in the serosal and mucosal compartments, respectively. Tissues were held under voltage-clamp conditions (0 V) and allowed to equilibrate for 20 min. Net electrogenic ion transport across the epithelium was recorded as short-circuit current (Isc; μA/cm^2^). The measurement of transepithelial potential and Isc allowed calculation of transepithelial electrical resistance (TER; Ω/cm^2^) according to Ohm’s law. Changes in net electrogenic ion flux were evaluated by measuring changes in Isc in response to neuronal depolarization with veratridine (10 μM serosal side; Calbiochem, 676950, San Diego, CA, USA) or in response to muscarinic receptor stimulation with carbachol (100 μM serosal side; C4382, Sigma-Aldrich). The difference between basal Isc and peak Isc recorded after veratridine or carbachol addition was measured (ΔIsc; μA/cm^2^). A positive ΔIsc indicated a luminally directed negative net charge transfer (anion secretion). Measurements were conducted and averaged in two adjacent ileal segments from the same mouse.

### Small intestine relaxation assay

The small intestine was removed and measured. The tissue was then placed in a 1 μM nifedipine solution for 10 min, after which the length of the small intestine was measured again and the variation (%) in length was determined.

### Quantification of fecal bacterial load

Fecal pellets (1–3) were collected in a sterile tube and fixed immediately on dry ice. Samples were stored at − 80 °C until analysis. DNA was extracted using a DNeasy PowerSoil kit (Qiagen, Toronto, ON, Canada) according to the manufacturer’s instructions, with minor modifications. Briefly, sample homogenization was done applying 30 Hz for 1 min in the TissueLyzer LT (Qiagen) and eluted in nuclease-free water. DNA load was quantified using NanoDrop (ThermoFisher Scientific) and standardized to 1 ng/μL in all samples. Next, a qPCR reaction was carried out using PerfeCTa SYBR Green SuperMix (QuantaBio, Beverly, MA, USA). Universal bacterial primers UniF340 and UniR514 [84] were used to amplify bacterial DNA. The qPCR mixture solution consisted of 10 μL reactions containing: 5 μL of master mix, 0.5 μL of 5 mM UniF340, 0.5 μL of 5 mM UniR514, 2 μL of nuclease-free water, and 2 μL of sample. The qPCR thermocycler (StepOnePlus; Applied Biosystems, Foster City, CA, USA) was set for an initial step at 95 °C for 3 min, followed by 40 cycles of 10 s at 95 °C and 45 s at 63 °C. Data were acquired in the final step at 63 °C. A melting curve was acquired at the end of the run. Results were calculated based on a standard curve generated with bacterial genomic DNA from a pure bacterium colony of *Clostridium sporogeneses*.

### Statistical analysis

Data is presented as mean ± standard error of the mean (SEM). Shapiro–Wilk’s and Kolmogorov–Smirnov’s tests were used to assess data normality. Student’s *t* test was applied for analysis between two groups. For multiple comparisons, we used one-way ANOVA, followed by Tukey’s multiple comparison test. Analyses of two variables were done with Two-way ANOVA, followed by either Tukey’s or Sidak’s multiple comparison test. The specific statistical test used in each figure panel is described in the figure legends. *n* is variable among experiments and described in every figure legend. Bar graphs display biological replicates as individual dots. *p* < 0.05 was accepted as statistically significant. Statistical analyses were performed with GraphPad Prism 8 (GraphPad Software, La Jolla, CA, USA).

## Supplementary Information

**Additional file 1: Figure S1.** Variation in small intestine length after muscle relaxant treatment *ex vivo*. Small intestine was removed from male mice and placed in a solution containing nifedipine (1 μM) for 10 minutes. (a) Small intestinal length variation before and after nifedipine treatment. **p<0.01, ***p<0.001, Student’s *t* test comparing Control vs Abx. (b) Small intestine length relative variation after nifedipine; *p*>0.05, Student’s *t* test. Data in panel b are expressed as mean ± SEM. *N*=4-5.

**Additional file 2: Figure S2.** Antibiotic (Abx) treatment induces a reduction in the number of enteric neurons. Representative immunofluorescent images of ganglia in the submucosal and myenteric plexuses in the colon: (**a**) HuC/D^+^ (green) and nNOS^+^ (magenta) neurons; (**b**) HuC/D^+^ (green) and ChAT^+^ (magenta) neurons. Scale bar: 30 μm.

**Additional file 3: Figure S3.** Reduction in Tuj1^+^ neuronal fibers and CALR^+^ neurons is observed after depletion of gut bacteria (**a**) Representative immunofluorescent images of ganglia in the submucosal and myenteric plexuses: Tuj1^+^ (green) neuronal fibers in the ileum and colon of control and antibiotic (Abx)-treated mice. Scale bar: 30 μm. (**b**) Quantification of Tuj1^+^ neuronal fibers in both ileal and colonic submucosal and myenteric plexuses. (**c**) Representative immunofluorescent images of ganglia in the submucosal and myenteric plexuses: calretinin (CALR)^+^ (magenta) neurons in the ileum and colon of control and antibiotic (Abx)-treated mice. Scale bar: 30 μm. (**d**) Number of CALR^+^ neurons in both ileal and colonic submucosal and myenteric plexuses. Data are expressed as mean ± SEM. n=3-5. **p<0.01, ***p<0.001; Student’s *t* test.

**Additional file 4: Figure S4.** Overlapping of PLP1 and S100B expression in enteric glial cells. Representative images of immunofluorescence in the colon of PLP1-eGFP mice stained with anti-S100B (white) antibody. Scale bar: 100 μm.

**Additional file 5: Figure S5.** Proliferative markers are not present in the ENS of either control or antibiotic (Abx)-treated mice. (**a**) Representative immunofluorescent images of sections of the ileum and the colon: HuC/D^+^ neurons (red), PLP1^+^ glia (green), Ki67^+^ proliferating cells (gray), merge with DAPI (blue) background staining. Scale bar: 50 μm. (**b**) Representative immunofluorescent images of sections of the ileum and the colon: HuC/D^+^ neurons (red), PLP1^+^ glia (green), EdU^+^ proliferating cells (gray), merge with DAPI (blue) background staining. Scale bar: 50 μm.

**Additional file 6: Figure S6.** Fecal bacterial load returns to control levels after antibiotic (Abx) withdrawal. Data are expressed as mean ± SEM. *n*=3-5. n.d.: not detectable.

**Additional file 7: Figure S7.** Spontaneous microbiota recolonization restores antibiotic (Abx)-induced enteric neuronal loss. Immunofluorescent images of representative ganglia of the submucosal and myenteric plexuses in the colon: HuC/D^+^ (green) and nNOS^+^ (magenta) neurons. Scale bar: 30 μm.

**Additional file 8: Figure S8.** Administration of the full concentration of metronidazole at the start of the experiment leads to body weight loss. Adult male mice were treated with a combination of antibiotics for 14 days in the drinking water (Abx group). The antibiotic regimen consisted of ampicillin (1 g/L), neomycin (1 g/L), vancomycin (0.5 g/L), and metronidazole (1 g/L). Graph shows body weight variation over the course of the 14-day experiment. Data are expressed as mean ± SEM. *n*=8. ***p<0.001; two-way ANOVA, followed by Sidak’s multiple comparison test.

## Data Availability

All data needed to evaluate the conclusions in the paper are present in the paper. Additional data related to this paper may be requested from the authors.

## Abbreviations

5-HT: Serotonin
Abx: Antibiotic
EGC: Enteric glial cell
ENS: Enteric nervous system
FSA: Fluorescein-5-6-sulfonic acid
GI: Gastrointestinal
LPS: Lipopolysaccharide
MAMPs: Microbial-associated molecular patterns
SCFA: Short-chain fatty acid
TER: Transepithelial electrical resistance
TLR: Toll-like receptor
