# Supplementary figures and images for: Intestinal microbiota shapes gut physiology and regulates enteric neurons and glia
Source: Microbiome. 2021 Oct 26;9:210. doi: 10.1186/s40168-021-01165-z (PMC8549243; doi:10.1186/s40168-021-01165-z)

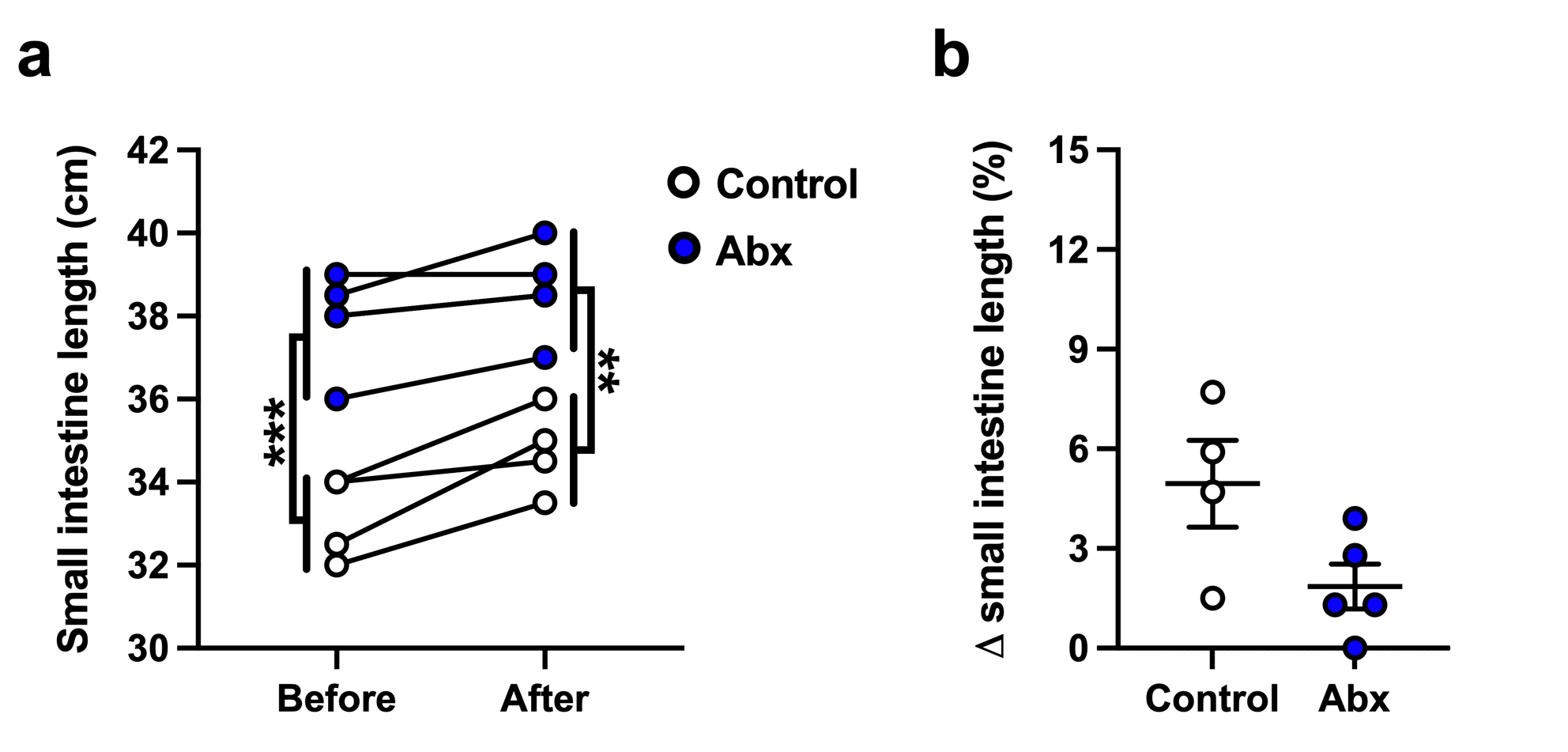

Supplement: Supplementary file 2 — Additional file 1: Figure S1. Variation in small intestine length after muscle relaxant treatment ex vivo. Small intestine was removed from male mice and placed in a solution containing nifedipine (1 μM) for 10 minutes. (a) Small intestinal length variation before and after nifedipine treatment. **p<0.01, ***p<0.001, Student’s t test comparing Control vs Abx. (b) Small intestine length relative variation after nifedipine; p>0.05, Student’s t test. Data in panel b are expressed as mean ± SEM. N=4-5. [file 40168_2021_1165_MOESM2_ESM.tiff]

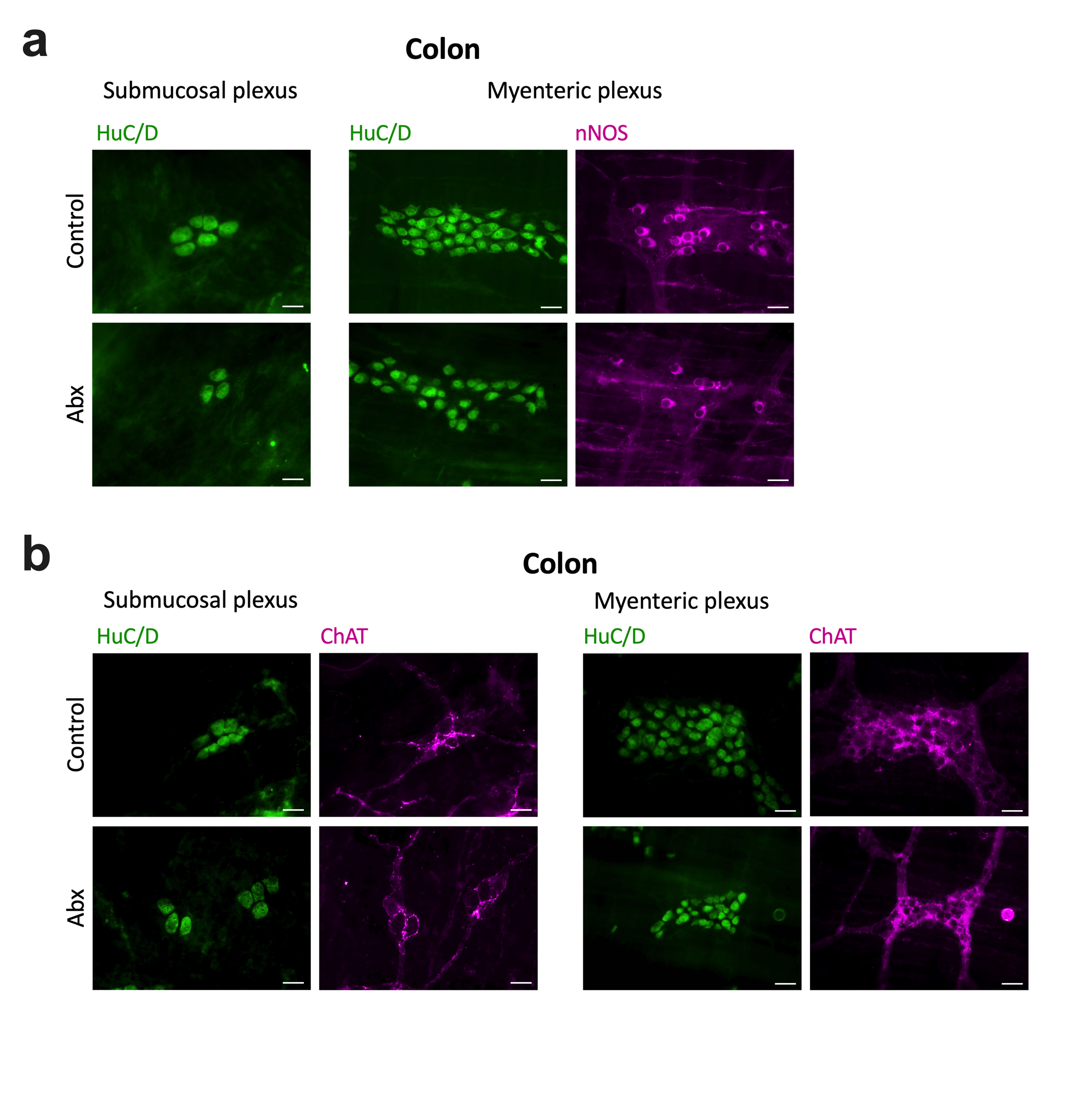

Supplement: Supplementary file 3 — Additional file 2: Figure S2. Antibiotic (Abx) treatment induces a reduction in the number of enteric neurons. Representative immunofluorescent images of ganglia in the submucosal and myenteric plexuses in the colon: (a) HuC/D+ (green) and nNOS+ (magenta) neurons; (b) HuC/D+ (green) and ChAT+ (magenta) neurons. Scale bar: 30 μm. [file 40168_2021_1165_MOESM3_ESM.tiff]

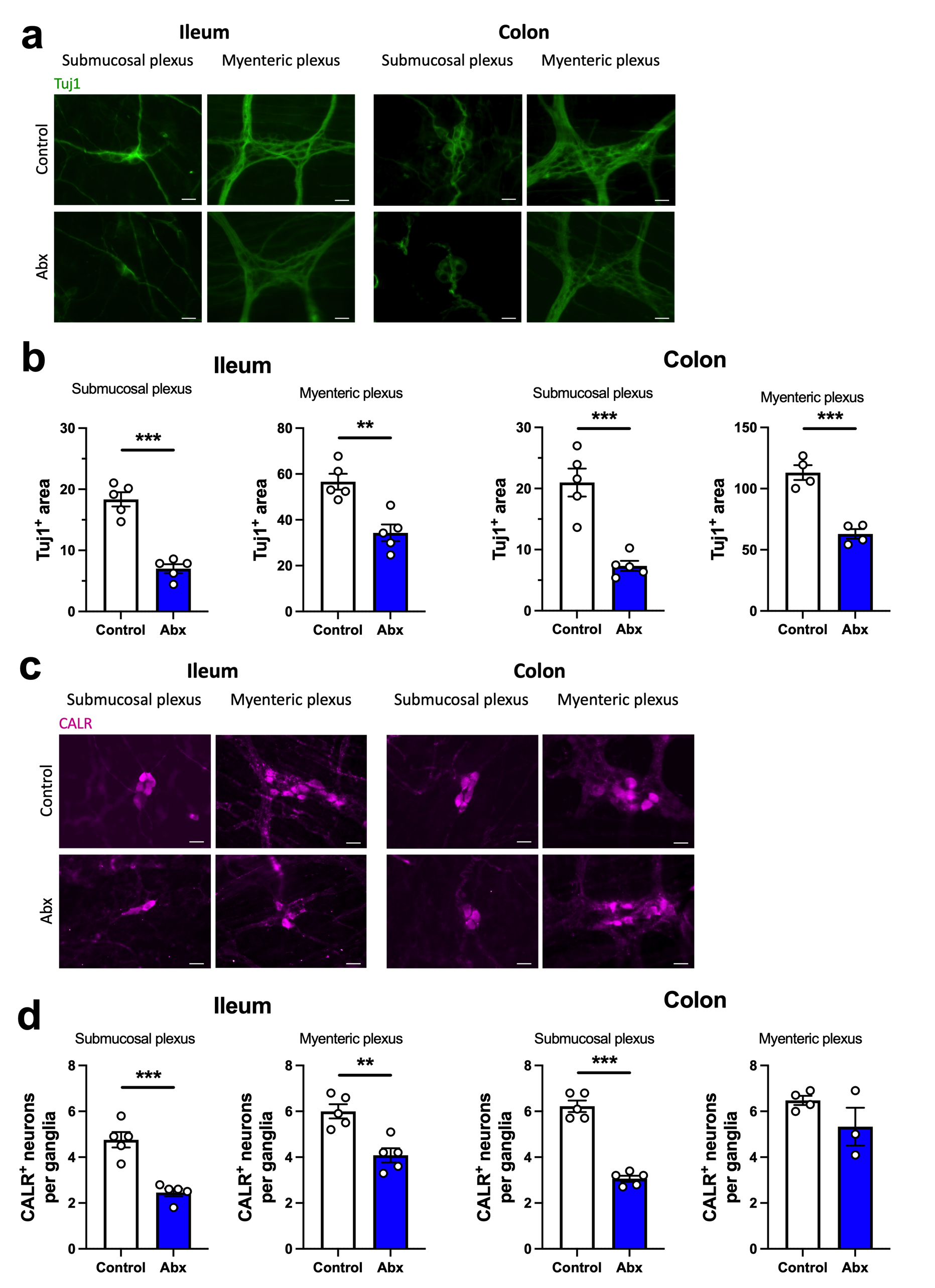

Supplement: Supplementary file 4 — Additional file 3: Figure S3. Reduction in Tuj1+ neuronal fibers and CALR+ neurons is observed after depletion of gut bacteria (a) Representative immunofluorescent images of ganglia in the submucosal and myenteric plexuses: Tuj1+ (green) neuronal fibers in the ileum and colon of control and antibiotic (Abx)-treated mice. Scale bar: 30 μm. (b) Quantification of Tuj1+ neuronal fibers in both ileal and colonic submucosal and myenteric plexuses. (c) Representative immunofluorescent images of ganglia in the submucosal and myenteric plexuses: calretinin (CALR)+ (magenta) neurons in the ileum and colon of control and antibiotic (Abx)-treated mice. Scale bar: 30 μm. (d) Number of CALR+ neurons in both ileal and colonic submucosal and myenteric plexuses. Data are expressed as mean ± SEM. n=3-5. **p<0.01, ***p<0.001; Student’s t test. [file 40168_2021_1165_MOESM4_ESM.tiff]

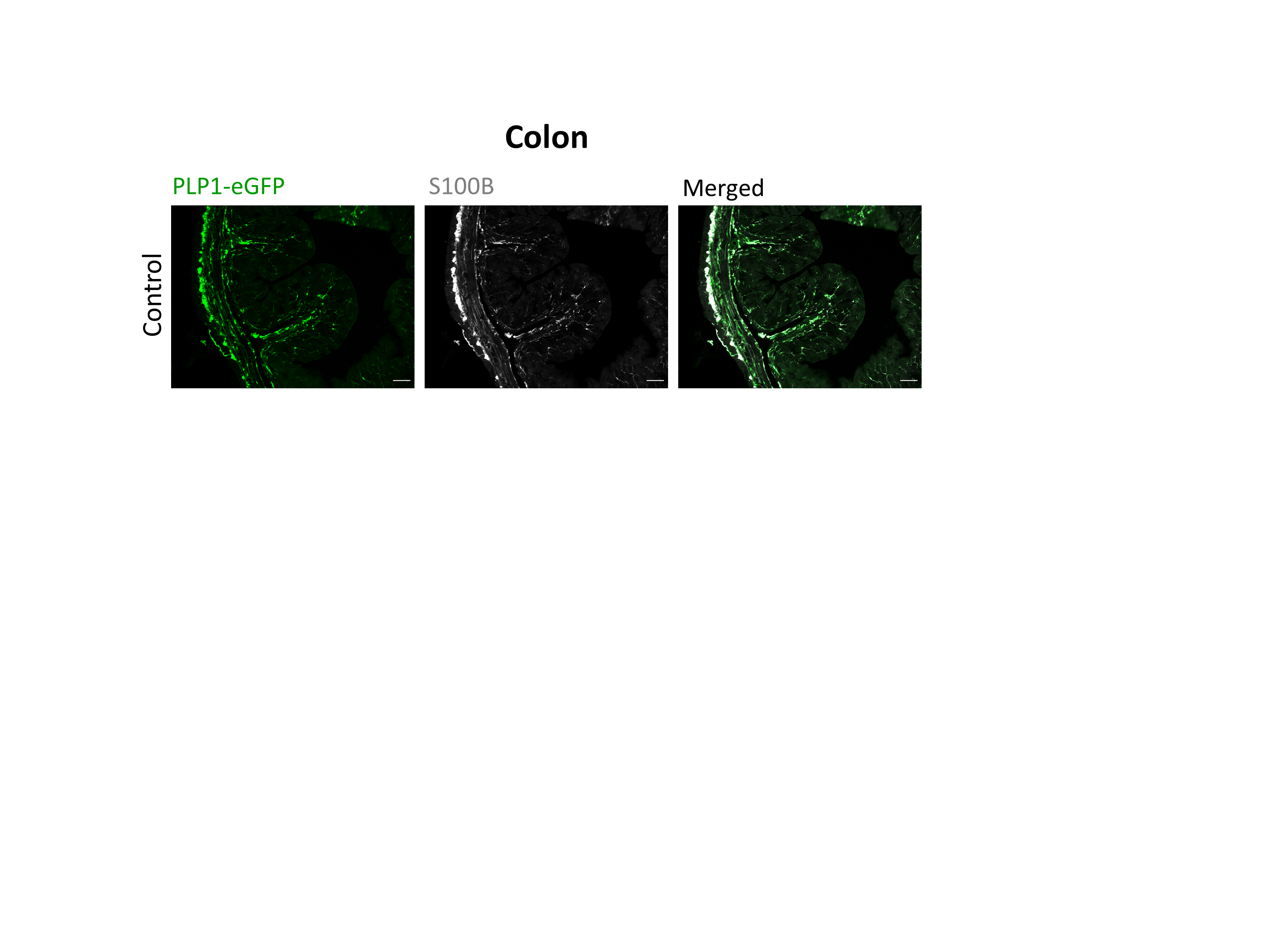

Supplement: Supplementary file 5 — Additional file 4: Figure S4. Overlapping of PLP1 and S100B expression in enteric glial cells. Representative images of immunofluorescence in the colon of PLP1-eGFP mice stained with anti-S100B (white) antibody. Scale bar: 100 μm. [file 40168_2021_1165_MOESM5_ESM.tif]

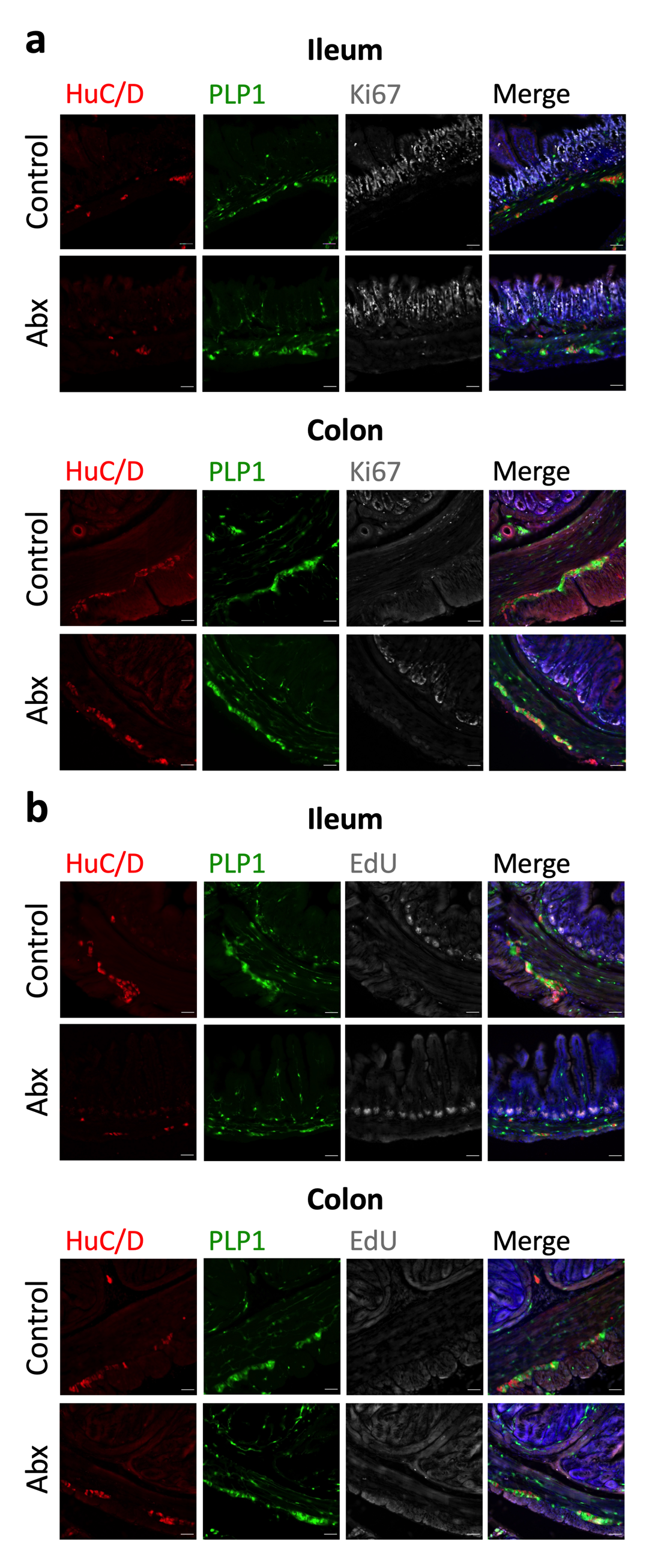

Supplement: Supplementary file 6 — Additional file 5: Figure S5. Proliferative markers are not present in the ENS of either control or antibiotic (Abx)-treated mice. (a) Representative immunofluorescent images of sections of the ileum and the colon: HuC/D+ neurons (red), PLP1+ glia (green), Ki67+ proliferating cells (gray), merge with DAPI (blue) background staining. Scale bar: 50 μm. (b) Representative immunofluorescent images of sections of the ileum and the colon: HuC/D+ neurons (red), PLP1+ glia (green), EdU+ proliferating cells (gray), merge with DAPI (blue) background staining. Scale bar: 50 μm. [file 40168_2021_1165_MOESM6_ESM.tiff]

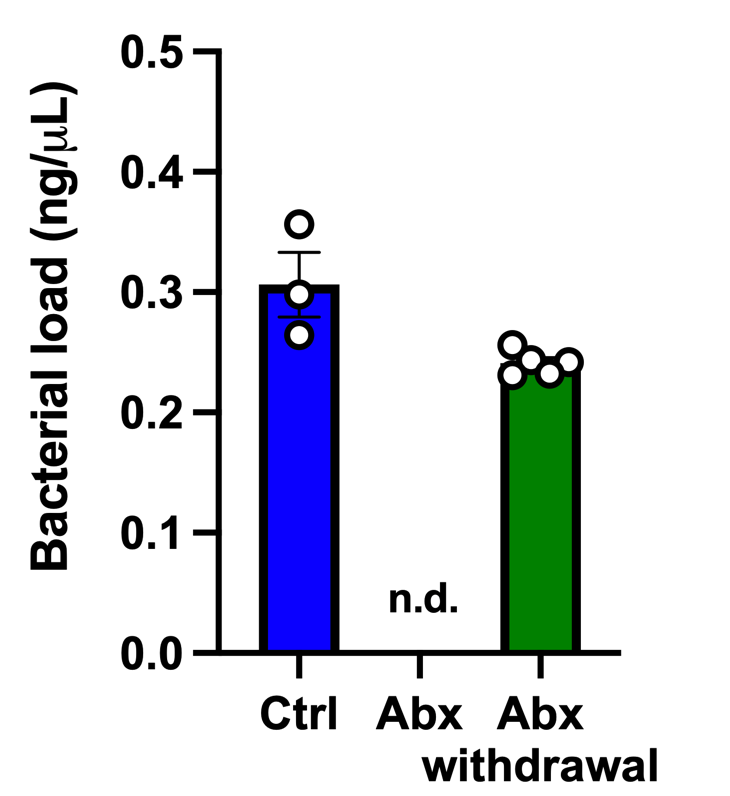

Supplement: Supplementary file 7 — Additional file 6: Figure S6. Fecal bacterial load returns to control levels after antibiotic (Abx) withdrawal. Data are expressed as mean ± SEM. n=3-5. n.d.: not detectable. [file 40168_2021_1165_MOESM7_ESM.tiff]

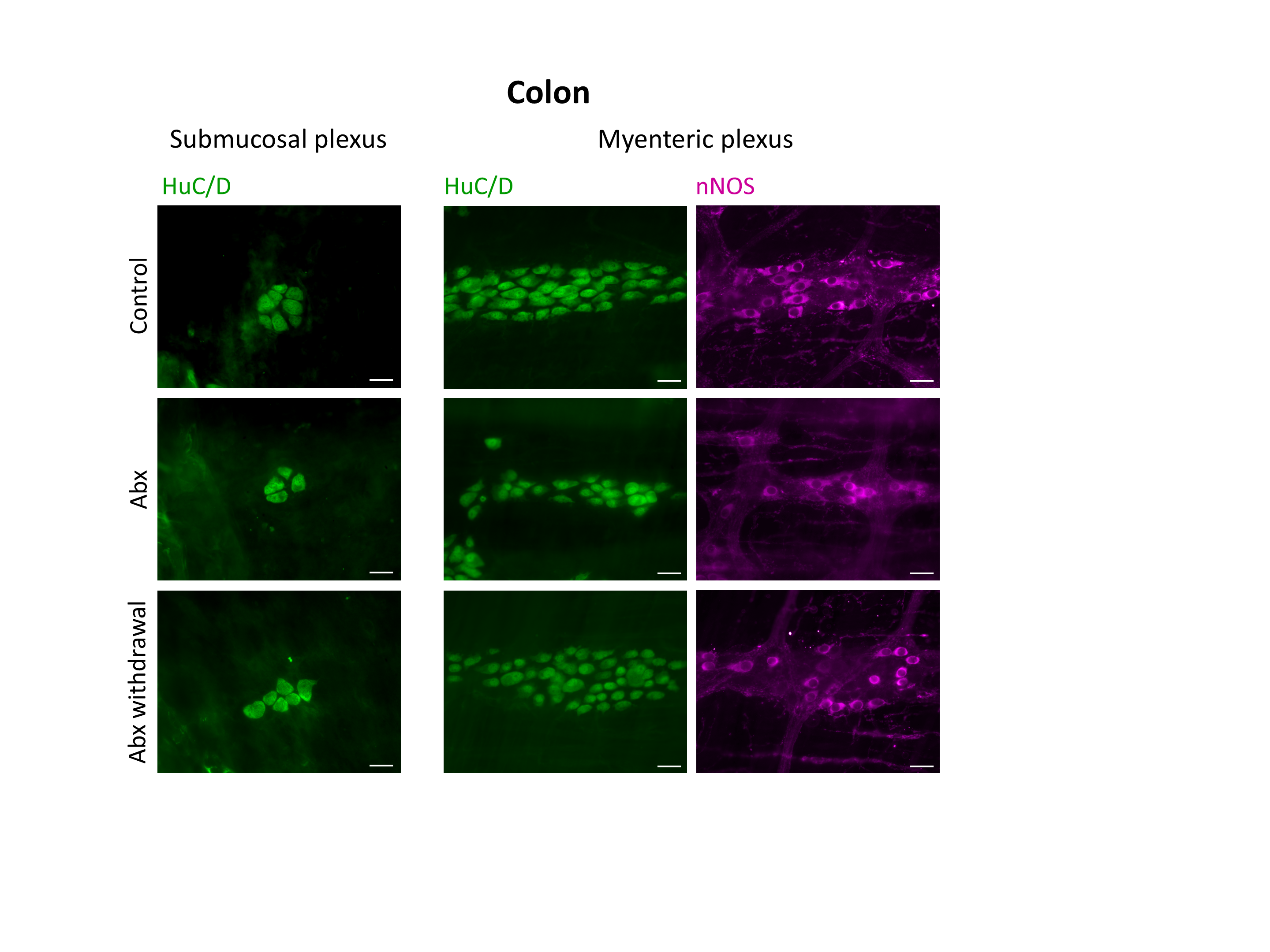

Supplement: Supplementary file 8 — Additional file 7: Figure S7. Spontaneous microbiota recolonization restores antibiotic (Abx)-induced enteric neuronal loss. Immunofluorescent images of representative ganglia of the submucosal and myenteric plexuses in the colon: HuC/D+ (green) and nNOS+ (magenta) neurons. Scale bar: 30 μm. [file 40168_2021_1165_MOESM8_ESM.tif]

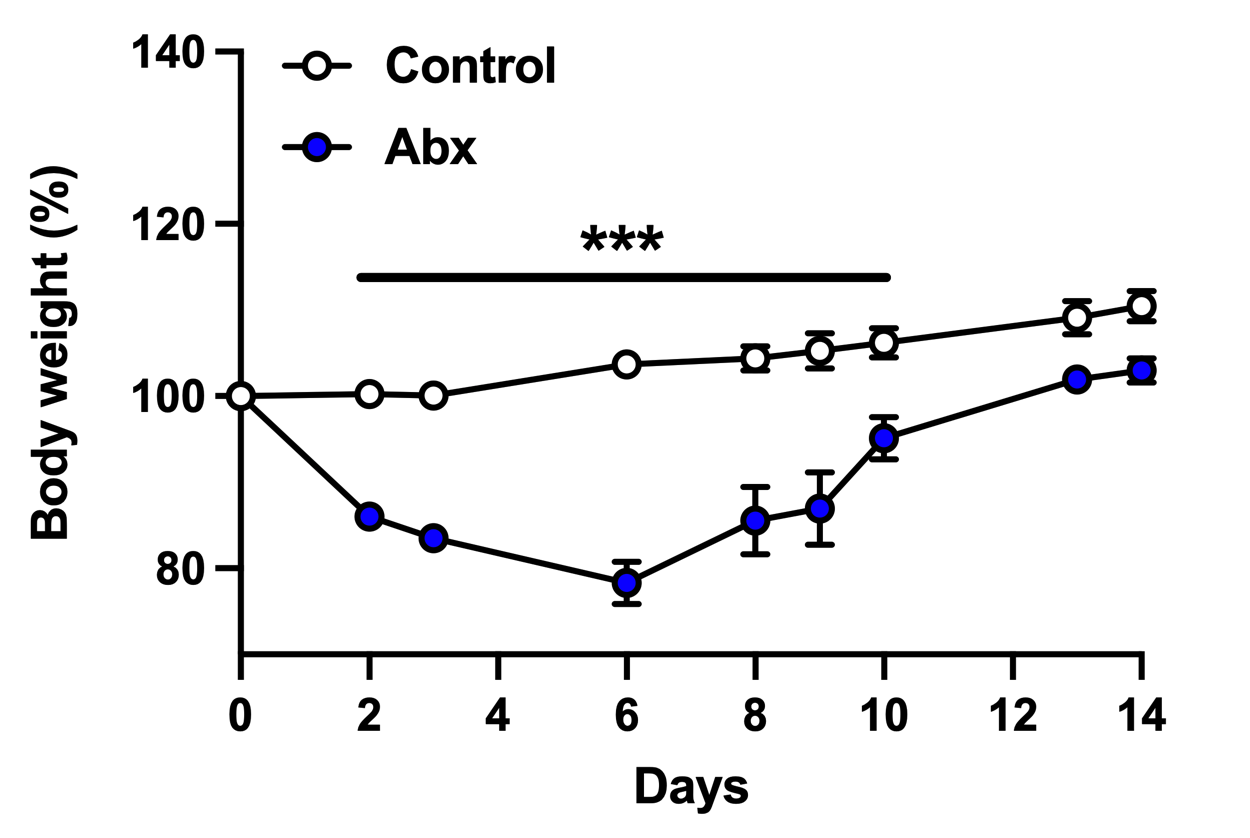

Supplement: Supplementary file 9 — Additional file 8: Figure S8. Administration of the full concentration of metronidazole at the start of the experiment leads to body weight loss. Adult male mice were treated with a combination of antibiotics for 14 days in the drinking water (Abx group). The antibiotic regimen consisted of ampicillin (1 g/L), neomycin (1 g/L), vancomycin (0.5 g/L), and metronidazole (1 g/L). Graph shows body weight variation over the course of the 14-day experiment. Data are expressed as mean ± SEM. n=8. ***p<0.001; two-way ANOVA, followed by Sidak’s multiple comparison test. [file 40168_2021_1165_MOESM9_ESM.tiff]
